# Regulation of biofilm gene expression by DNA replication in *Bacillus subtilis*

**DOI:** 10.1111/jcmm.18481

**Published:** 2024-06-20

**Authors:** Renjie Wu, Ling‐Xing Kong, Feng Liu

**Affiliations:** ^1^ National Laboratory of Solid State Microstructures, Department of Physics, Collaborative Innovation Center of Advanced Microstructures and Institute for Brain Sciences Nanjing University Nanjing P. R. China

## Abstract

*Bacillus subtilis* relies on biofilms for survival in harsh environments. Extracellular polymeric substance (EPS) is a crucial component of biofilms, yet the dynamics of EPS production in single cells remain elusive. To unveil the modulation of EPS synthesis, we built a minimal network model comprising the SinI‐SinR‐SlrR module, Spo0A, and EPS. Stochastic simulations revealed that antagonistic interplay between SinI and SinR enables EPS production in bursts. SlrR widens these bursts and increases their frequency by stabilizing SinR‐SlrR complexes and depleting free SinR. DNA replication and chromosomal positioning of key genes dictate pulsatile changes in the *slrR*:*sinR* gene dosage ratio (*g*_r_) and Spo0A‐P levels, each promoting EPS production in distinct phases of the cell cycle. As the cell cycle lengthens with nutrient stress, the duty cycle of *g*_r_ pulsing decreases, whereas the amplitude of Spo0A‐P pulses elevates. This coordinated response facilitates keeping a constant proportion of EPS‐secreting cells within colonies across diverse nutrient conditions. Our results suggest that bacteria may ‘encode’ *eps* expression through strategic chromosomal organization. This work illuminates how stochastic protein interactions, gene copy number imbalance, and cell‐cycle dynamics orchestrate EPS synthesis, offering a deeper understanding of biofilm formation.

## 1 INTRODUCTION

*Bacillus subtilis* is a model organism for exploring cell fate decision. It can transition among various cell states to adapt to environmental fluctuations.^1^, ^2^ Under mild nutrient deficiency, for example, free‐swimming cells can synthesize and secrete extracellular polymeric substance to form a biofilm.^3^ Within the biofilm, bacteria engage in continuous transport and exchange of nutrients, air, and small molecules to achieve more efficient nutrient acquisition, enhance metabolic capabilities, and develop resistance to antibiotics.^4^, ^5^, ^6^ Although key players in biofilm formation have been identified,^7^, ^8^, ^9^ the precise orchestration of its components is not fully understood.

The regulation of EPS synthesis primarily involves a phosphorelay module controlling the level of phosphorylated Spo0A (Spo0A‐P) and a downstream three‐protein module comprising SinI, SinR and SlrR. With sufficient nutrient availability, the transcriptional repressor SinR is constitutively expressed and maintained at relatively high levels, inhibiting the expression of genes involved in EPS synthesis such as *epsA‐O*.^8^, ^10^, ^11^, ^12^ Under nutrient limitation, the bacterial growth rate slows down, leading to gradual accumulation of phosphorelay proteins (e.g. phosphorylated KinA) and upregulation of Spo0A‐P.^13^, ^14^ Typically, the Spo0A‐P level exhibits pulses, with the amplitude increasing with enhanced nutrient deficiency.^13^ As a master regulator, Spo0A‐P induces the expression of *sinI* and *sinR*.^15^, ^16^ SinI can form a stable heterodimer with SinR,^17^, ^18^, ^19^ sequestering its transcriptional activity and promoting the production of EPS and SlrR.^11^, ^20^ Meanwhile, SlrR also dimerizes with SinR to deplete free SinR,^17^, ^21^ forming a positive feedback loop (PFL).

By deleting *slrR* and decoupling *sinI* from its known regulators via an IPTG‐inducible construct, Lord et al. found that activation of the genetic switch controlling EPS production could depend solely on antagonistic interplay between SinR and SinI.^22^ In this setup, the switching frequency is governed by the ratio (*r*) of SinI to SinR synthesis rates. It can be inferred from Ref. 23 that a larger *r*, achieved by upregulating Spo0A‐P abundance, would increase the switching rate and potentially boost the proportion of EPS‐secreting cells in a colony. But this speculation contradicts the observed stable EPS secretion (around 25% of cells in NCIB 3610 colonies^24^) under nutrient‐limited conditions, even persisting for three days.^25^ This discrepancy suggests the presence of a yet‐to‐be‐identified mechanism that mitigates the effect of increasing the Spo0A‐P pulsing amplitude.

Notably, Spo0A‐P pulsing follows the pulsing of the *kinA*:*spo0F* gene dosage ratio induced by DNA replication,^14^ and the functioning of SinR and SinI is sensitive to their gene dosage,^7^, ^8^, ^9^ but the underlying mechanism remains unclear. It seems essential to take into account the impact of DNA replication on the switch dynamics and EPS synthesis. Indeed, the transient copy number imbalance, where genes before and after the replication fork have distinct copies for a while, can modulate cell signalling. For instance, copy number variation can influence cell fate or coordinate interlinked processes.^26^ Remarkably, the *sinR* and *slrR* genes are located far apart on the chromosome, which could have functional implications for their dosage‐dependent regulation.

The current study aims to unravel the regulation of EPS synthesis across diverse nutrient conditions, focusing on the influence of stochastic protein antagonism and chromosomal arrangement of *sinR* and *slrR* on EPS production. We developed a minimal network model, consisting of the SinI‐SinR‐SlrR module, Spo0A‐P and EPS. Given the high abundance of proteins in the complex phosphorelay module (e.g. 600–2000 Spo0A proteins per cell in starvation and 750–1500 Spo0F proteins per cell), resulting in minimal intrinsic noise, we employed ordinary differential equations (ODEs) to simulate Spo0A‐P pulses as inputs to the SinI‐SinR‐SlrR module. Conversely, to accurately represent noise within the SinI‐SinR‐SlrR module, particularly stochastic fluctuations in free SinR that regulate EPS production, we utilized the standard Gillespie algorithm for stochastic simulations. EPS production occurs in bursts, with intermittent sharp increases in molecule numbers. We explored the distributions for inter‐burst interval and burst width and the proportion of EPS‐expressing cells in a colony under diverse conditions. The SinR‐SlrR PFL, gene copy number variation during DNA replication, and pulsatile Spo0A‐P dynamics are coordinated to dictate the switch and EPS expression, allowing bacteria to transition into the biofilm state at different growth rates.

## 2 MATERIALS AND METHODS

### 2.1 Model

**FIGURE 1:**
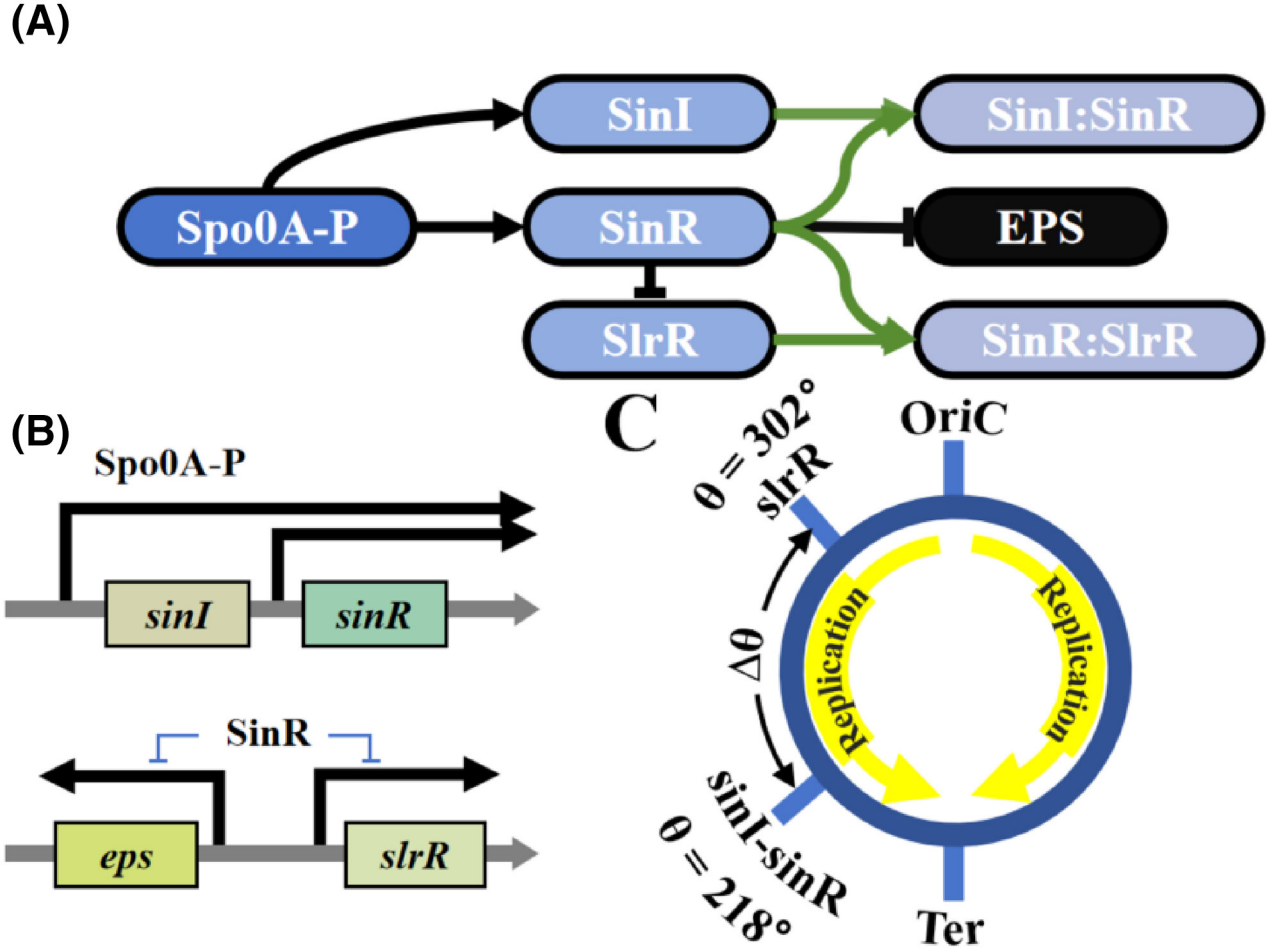
Schematic of the network model. (A) Model of the network controlling EPS synthesis. Black lines with triangular arrows and T‐bar represent transcriptional activation and inhibition, respectively, while green lines denote the protein dimerization. (B) Spo0A‐P activates the transcription of *sinI* and *sinR*, while SinR suppresses the transcription of *eps* and *slrR*. The arrows represent the direction of transcription. (C) Locations of certain gene loci on the chromosome. *sinI* and *sinR* are in the same operon, whereas *slrR* is far from them. The replication forks move from the origin (OriC) to the terminus (Ter).

Since the *sinI* and *sinR* genes are located in the same operon, Spo0A‐P activates their synthesis simultaneously^27^ (Figure 1A,B). SinR forms dimers with SinI or SlrR, suppressing its own transcriptional activity. These heterodimers are highly stable,^17^ whose dissociation is negligible. The following processes are included in the model:

**Table:** 

$\overset{k_{I}}{\to }nI$	$I\overset{d_{I}}{\to }\emptyset$
$\overset{k_{R}}{\to }nR$	$R\overset{d_{R}}{\to }\emptyset$
$\overset{k_{\mathrm{rR}}}{\to }n\mathrm{rR}$	$\mathrm{rR}\overset{d_{\mathrm{rR}}}{\to }\emptyset$
$I+R\overset{k_{\text{IR}}}{\to }\mathrm{IR}$	$\mathrm{IR}\overset{d_{\mathrm{IR}}}{\to }\emptyset$
$\mathrm{rR}+R\overset{k_{\text{RrR}}}{\to }\mathrm{RrR}$	$\mathrm{RrR}\overset{d_{\mathrm{RrR}}}{\to }\emptyset$
$\overset{k_{Z}}{\to }nZ$	$Z\overset{d_{z}}{\to }\emptyset$

I, R, rR, IR and RrR represent the SinI, SinR and SlrR proteins, as well as the SinI:SinR and SinR:SlrR dimers, respectively. Z denotes biofilm‐associated proteins, exemplified by EPS, and ø indicates degradation. The rate constant of each reaction is marked above the arrow and is detailed in Table S1. *d*_i_ (*i* = I, R, rR, IR, RrR or Z) signifies the degradation rate constant, while *k*_IR_ and *k*_RrR_ are the rate constants for the formation of IR and RrR heterodimers, respectively.

Since stochastic fluctuations caused by antagonistic protein–protein interactions (SinR‐SinI and SinR‐SlrR) drive the genetic switching, we need consider the stochasticity of gene expression. Each mRNA species is produced with rate *k*_i_ (*i* = I, R, rR or Z). As the lifetime of mRNA molecules is exceedingly short,^28^ individual mRNA molecules rapidly degrade after generating a burst of protein molecules. The temporal interval between two successive bursts occurring at *t*_0_ and *t*_0_ + *τ* follows the distribution $\;f_{i}\left(\tau \right)=k_{i}\left(t_{0}+\tau \right)e^{-\int_{t_{0}}^{t_{0}+\tau }k_{i}\left(t^{\prime }\right)dt^{\prime }}$. In accordance with the experimental and theoretical studies,^29^, ^30^, ^31^ the probability of producing *n* molecules per burst is

(1) $$P_{i}\left(n\right)=\frac{b_{i}^{n}}{{\left(b_{i}+1\right)}^{n+1}},$$

where *b*_*i*_ denotes the mean burst size.

Hill functions are widely used to model mRNA production rates under transcriptional control.^32^, ^33^, ^34^ Meanwhile, the rates of gene expression in our model are assumed to be proportional to the gene copy number and influenced by the cell growth rate (*μ*). SinI and SinR are induced primarily by Spo0A‐P, and the corresponding rate *k*_i_ is

(2) $$k_{i}=\frac{g_{i}\left(t\right)}{F\left(\mu \right)}\left[\lambda_{i}\frac{m{\left(t\right)}^{n_{i}}}{m{\left(t\right)}^{n_{i}}+K_{i}^{n_{i}}}+\Delta \lambda_{i}\right].$$

SlrR and Z are repressed by SinR, and the rate *k*_i_ is

(3) $$k_{i}=\frac{g_{i}\left(t\right)}{F\left(\mu \right)}\lambda_{i}\frac{K_{i}^{n_{i}}}{{\left[R\right]}^{n_{i}}+K_{i}^{n_{i}}}.$$

*g*_i_ (*t*) denotes the copy number of gene *i* at time *t*, *λ*_i_ governs the strength of regulated expression, *m* is an intermediate variable controlled by the Spo0A‐P level, and [*R*] is the concentration of SinR. *F*(*μ*) is introduced to reflect the influence of cell growth on gene transcription.^35^, ^36^ To select the appropriate form of *F*(*μ*), we set it to 1 when the cell volume doubles every hour (i.e. *μ* = log2 h^−1^). Accordingly, we used the same phenomenological expression for *F*(*μ*) as in Ref. 13:

(4) $$F\left(\mu \right)=ae^{\mathrm{b\mu}}+c,$$

with *a* = 0.690, *b* = 0.689 and *c* = 0.745.

Of note, the *sinR* gene has three promoters that allow transcripts of different sizes —0.4 kb, 0.7 kb and 1.2 kb.^27^ The 0.4 kb transcript is specific to the *sinR* gene, while the other two transcripts are co‐transcribed with the *sinI* one. Thus, we introduced a constant difference in the basal transcription rate between *sinI* and *sinR* (i.e. ∆*λ*_I_ = 0 and ∆*λ*_R_ = ∆*λ*), while the same *g* and *λ* are used to describe their regulated synthesis rates of mRNAs.

The expression of *sinI* and *sinR* is regulated by Spo0A‐P both directly and indirectly (Spo0A‐P represses the synthesis of *σ* ^A^ and AbrB,^37^, ^38^ which in turn transcriptionally regulate *sinI* and *sinR*), involving the coherent feedforward loops. The cell growth has global effects on all gene expressions via protein dilution.^39^ Introduction of the intermediate variable is to model the delay caused by the indirect control and cell growth; its dynamics satisfy^13^

(5) $$\;\frac{\mathrm{dm}}{\mathrm{dt}}=\left(K_{s}+\mu \right)\;\left(S-m\right).$$

The dynamics of the Spo0A‐P level (*S*) are simulated using the same model as in Ref. 13 (see Data S1 for details).

The copy number of gene *i* (*g*_i_) depends on the progression of the cell cycle and its location on the chromosome, characterized by an angle *θ*_i_ = *D*_i_ × 360°/*L*, where *D*_i_ is the distance between the gene and replication origin and *L* is the chromosome length. In the wild‐type strain (NCIB 3610), *θ*_I_ = *θ*_R_ = *θ*_IR_ = 218°, *θ*_rR_ = 302° and *θ*_eps_ = 301°, that is, the angular difference (∆*θ*) between *sinI/R* and *slrR* equals 84° (Figure 1C). After replication initiation, the replication fork moves from OriC at 0° to Ter at 180°. *g*_i_ is 1 before DNA replication and jumps to 2 after replication in each cell cycle, which is described by a step function:

(6) $$g_{i}\left(t\right)=\left\{\begin{array}{c} 1\;0\leq t<t_{\mathrm{ri}}\\ 2\;t_{\mathrm{ri}}\leq t<T_{\text{cell}} \end{array}\right.,$$

where *t* represents the time elapsed since the last cell division (Figure S1). *T*_cell_ and *t*_ri_ are the cell cycle length and the moment when the replication fork reaches this gene, respectively. Given a uniform replication rate, *t*_ri_ = *T*_pre_ + *T*_rep_ (180°‐|180°‐*θ*_i_|)/180°, where *T*_pre_ represents the duration of the preparation phase preceding DNA replication, and *T*_rep_ signifies the total duration of the replication period. According to the experimental data and model in Refs. 12, 13, *T*_pre_ = 0.05*T*_cell_, *T*_rep_ = 0.78 + 0.15*T*_cell_/log2 if *T*_cell_ >1.5 h and *T*_rep_ = 0.95*T*_cell_ otherwise.

During rapid cell growth, multiple replication rounds can occur within one cell cycle, leading to *g* >2 for some genes.^40^ Here we considered only one replication event per cycle under nutrient deficiency. The ratio of *g*_rR_ to *g*_IR_, *g*_r_, periodically alternates between 1 and 2 in a stable nutrient environment (Figure S1).

### 2.2 Methods

We ran stochastic simulations using the standard Gillespie algorithm for species in the SinI‐SinR‐SlrR module, while *m* and *S* were characterized deterministically. *N* with subscripts represents the number of free molecules. The default parameter values are listed in Table S1, and the state update algorithm is presented in Table S2. All numerical simulations and data analysis were conducted using codes programmed in Python 3.9.13 and MATLAB 2021b. Unless explicitly stated, all time measurements are given in the number of cell cycles or generations. The cell volume satisfies $V\left(t\right)=V_{0}e^{\mathrm{\mu t}}$, where *V*_0_ is the volume after cell division. The numbers of proteins and cell volume are simply equally halved after cell division.

## 3 RESULTS

### 3.1 DNA replication modulates biofilm‐associated gene expression

**FIGURE 2:**
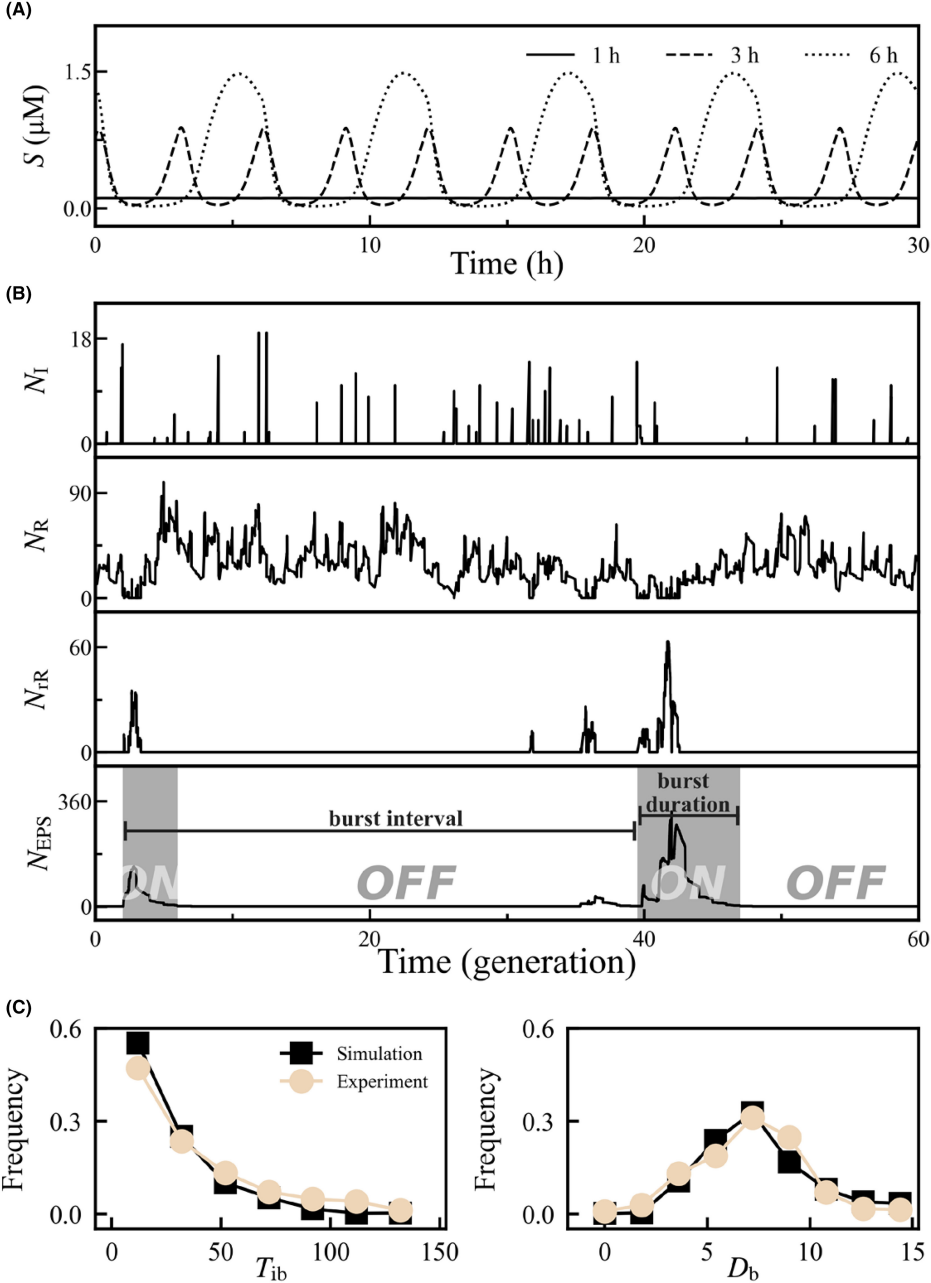
Overview of the input and network dynamics under DNA replication. (A) *S* (*t*) for *T*_cell_ = 1, 3, or 6 h. (B) Temporal evolution of *N*_I_, *N*_R_, *N*_rR_ and *N*_EPS_ (from top to bottom) on a single simulation trial with *T*_cell_ = 1 h (*μ =* log2 h^−1^). In the bottom panel, the bursts are marked in grey. A more detailed depiction of the dynamics of network components is presented in Figure S3. (C) Distribution of the burst interval (left) and burst duration (right) of EPS production with *T*_cell_ = 30 min (*μ =* 2log2 h^−1^). The experimental data in Ref. 22 are shown for comparison. In (B) and (C), the fluctuations of *S* are negligible, and *S* (*t*) simplifies to a constant of 0.11 μM.

According to the model in Ref. 13, the input *S*(*t*) (i.e. the Spo0A‐P level) and intermediate variable *m*(*t*) always oscillate in response to nutrient stress, with the period and amplitude determined by *T*_cell_ (Figure 2A and S2). For *T*_cell_ ≤1 h, these fluctuations are negligible, so *S*(*t*) and *m*(*t*) simplify to a constant of 0.11 μM in simulation. Since SinR strongly inhibits *eps* transcription,^12^ a scarcity of free SinR promotes EPS production, resulting in its burst‐like synthesis (Figure 2B and S3). Individual bursts can span multiple cell cycles (the method for identifying bursts is presented in Figure S4, S5); the inter‐burst interval (*T*_ib_) and burst duration (*D*_b_) reflect the activation frequency and ON‐time duration of the switch, respectively.

To validate our model, we mimicked the experimental protocol in Ref. 22 where only the SinI‐SinR module was considered (without SlrR production, i.e. *k*_rR_ = 0) and *T*_cell_ = 30 min. Substantial variability in both *T*_ib_ and *D*_b_ can be observed (Figure 2C); *T*_ib_ exhibits a near‐exponential distribution, while *D*_b_ displays a unimodal distribution, broadly consistent with the data in Ref. 22. The near‐exponential distribution of *T*_ib_ suggests a memoryless initiation of EPS expression, which is caused by noisy antagonism between SinR and its antagonists, SinI and SlrR. The unimodal distribution of *D*_b_ indicates a requirement for some degree of memory; this memory depends on both the continued production of EPS and the decay (degradation and dilution) of existing EPS molecules. Together, the noisy antagonism between SinI and SinR is essential for EPS bursting behaviour.

**FIGURE 3:**
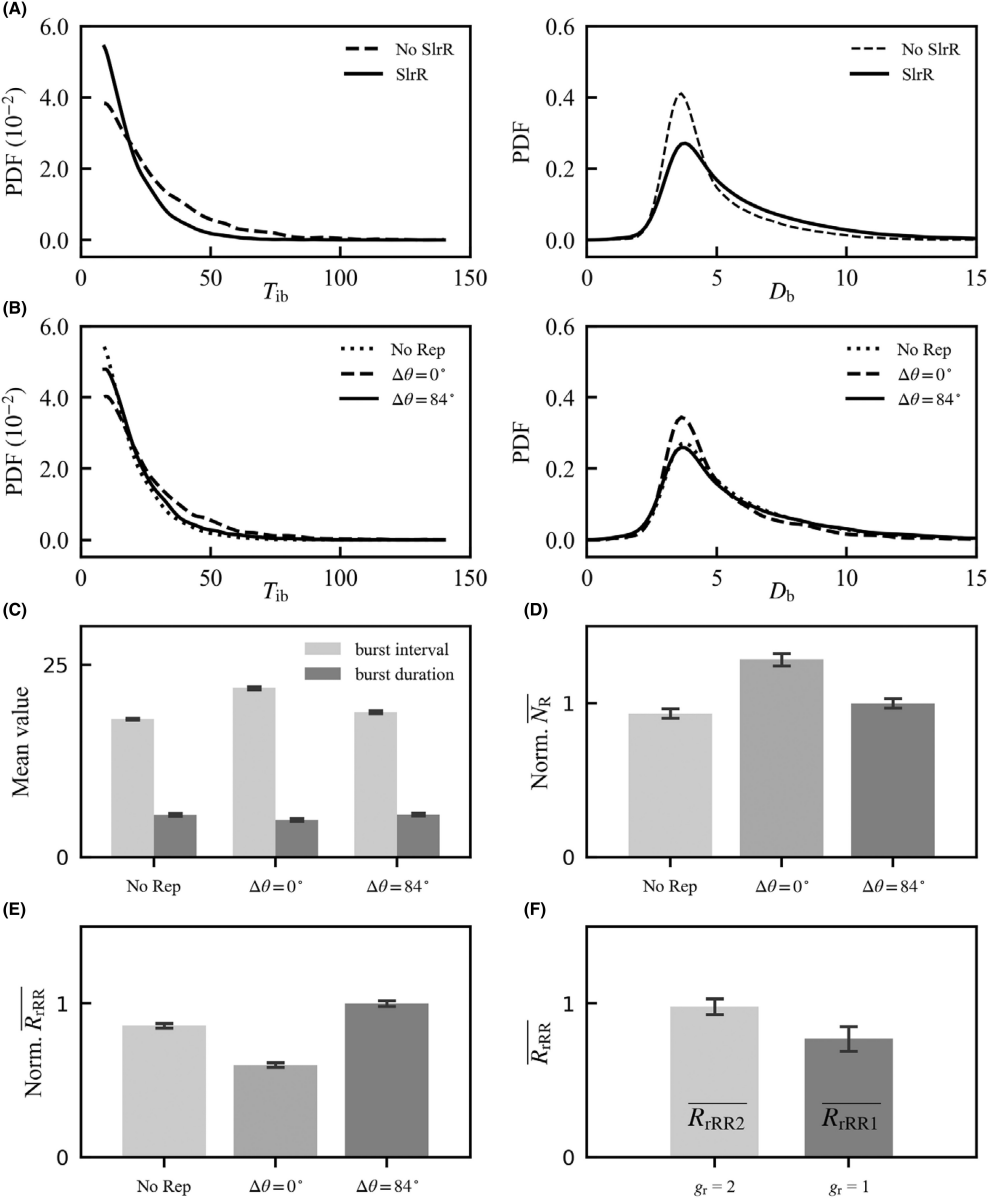
Roles for SlrR in promoting EPS production under DNA replication. (A) Probability density functions (PDFs) for *T*_ib_ (left) and *D*_b_ (right) in the presence or absence of SlrR without DNA replication. (B‐E) PDFs for *T*_ib_ (left) and *D*_b_ (right) (B), the mean values of *T*_ib_ and *D*_b_ (C), mean number of free SinR proteins ($\;\bar{N_{R}}$; D), and mean ratio of the total amount of SlrR to that of SinR ($\bar{R_{\mathrm{rRR}}}$; E) for three scenarios: no DNA replication, DNA replication with ∆*θ* =0° and ∆*θ* =84°. The means were obtained by averaging over a long period of 10,000 h. $\bar{N_{R}}$ and $\bar{R_{\mathrm{rRR}}}$ are normalized to the wild‐type case. (F) Average of *R*_rRR_ over the period with *g*_r_ =1 or *g*_r_ =2 under DNA replication and ∆*θ* =84°. The input *S*(*t*) is always set to 0.11 μM, and *T*_cell_ = 1 h. Error bars represent S.E.M.

We then explored the role for SlrR in EPS synthesis. Without DNA replication, the probability density function (PDF) for *T*_ib_ drops monotonically toward 0 with increasing *T*_ib_, while the PDF for *D*_b_ rises to a peak and then declines (Figure 3A). Compared with the SlrR‐free case, *T*_ib_ shows a narrower distribution with SlrR, while *D*_b_ has a wider spread and a higher mean. Thus, SlrR increases the average burst frequency and duration. Indeed, SlrR promotes depleting free SinR, facilitating e*ps* expression. Additionally, the SinR‐SlrR PFL can prolong the dwell time of SinR in its low‐level state, leading to larger *D*_b_. Taken together, the presence of SlrR facilitates widening EPS bursts and diminishing free SinR to boost the burst frequency.

When the *slrR* and *sinI‐sinR* genes are closely positioned (∆*θ* = 0° and *θ*_IR_ = 218° in a hypothetical scenario), DNA replication weakens EPS production compared with no replication, marked by lower averages of 1/*T*_ib_ and *D*_b_ (Figure 3B,C) and higher average *N*_R_ (Figure 3D). Notably, the total amounts of SinI and SinR (including dimers), *N*_Itot_ and *N*_Rtot_, change slightly over time due to balanced production and degradation rates, while the total amount of SlrR (*N*_rRtot_) is determined only by free SinR. The key factor here is the *N*_rRtot_:*N*_Rtot_ ratio (*R*_rRR_); its lowest mean at ∆*θ* = 0° (Figure 3E) reveals that DNA replication directly increases SinR abundance, enhancing its transcriptional inhibition of *slrR* and *eps*.

In the wild‐type case with ∆*θ* = 84°, the average of *T*_ib_ is slightly higher compared to the no‐replication case, while *D*_b_ remains similar (Figure 3C). This discrepancy stems from enhanced SinR production and a transient doubling of *g*_r_ during roughly half the replication period per cycle (*slrR* replicates before *sinR*). Each cell cycle can be divided into three phases, with *g*_r_ equaling 1, 2 and 1 respectively. Let $\bar{R_{\mathrm{rRR}1}}$ and $\bar{R_{\mathrm{rRR}2}}$ represents the average of *R*_rRR_ over phases 1 and 3 and phase 2, respectively. $\bar{R_{\mathrm{rRR}2}}$ is higher than $\bar{R_{\mathrm{rRR}1}}$ (Figure 3F), indicating that SlrR production is boosted during the *g*_r_ = 2 phase, which facilitates EPS synthesis. Collectively, the chromosomal arrangement of antagonistic genes enables regulation of *eps* expression through *g*_r_ pulsing.

**FIGURE 4:**
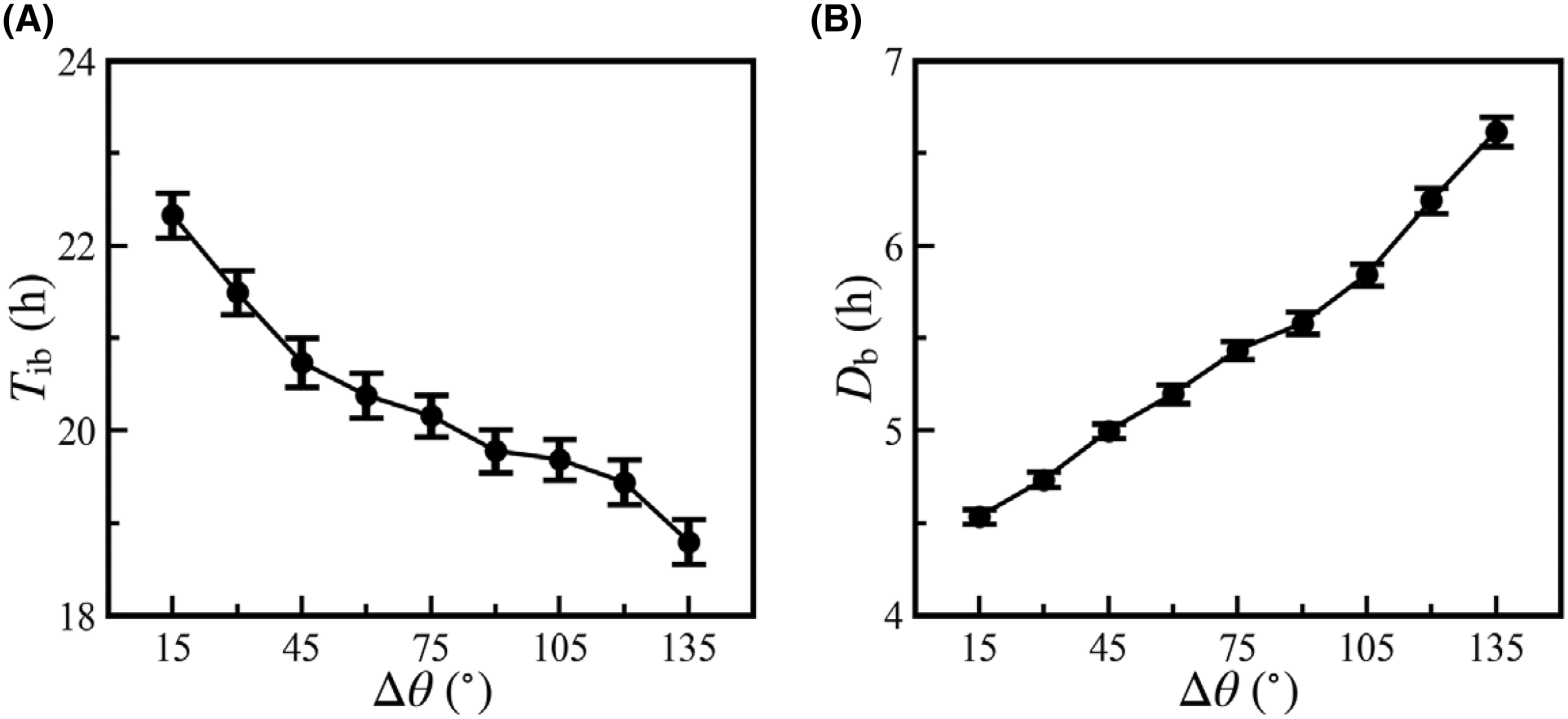
Influence of gene positioning on EPS dynamics. Mean values of *T*_ib_ (A) and *D*_b_ (B) versus the angular difference between *slrR* and *sinI‐sinR*. The input *S* is set to 0.11 μM, and *T*_cell_ = 1 h. An average over 600 trials was taken for each case. Error bars represent S.E.M.

It is worthy to systematically investigate the impact of Δ*θ* on EPS dynamics with constant input. Imagine diverse mutant strains with various Δ*θ* values, where the *sinR* gene remains at 218° and *slrR*'s location varies between 0° and 218°. As expected, increasing Δ*θ* boosts the average burst frequency and extends the burst duration (Figure 4). Given the fixed durations of the cell cycle and DNA replication, a larger Δ*θ* corresponds to a longer segment with *g*_r_ = 2 per cell cycle, that is, an increase in the duty cycle of *g*_r_ pulsing, promoting EPS production. Altogether, the switch hinges on SinI‐SinR antagonism; the SinR‐SlrR PFL primarily modulates the switching timing, while Δ*θ* controls the duty cycle of *g*_r_, shaping burst characteristics.

### 3.2 Impact of elongated cell cycle on EPS expression via DNA replication

As shown above, the SinR‐SlrR PFL and separation of *sinI‐sinR* from *slrR* are combined to promote EPS synthesis in *B. subtilis*. This effect is closely associated with the *g*_r_ pulsing, whose duty cycle is determined by both Δ*θ* and other factors such as the durations of DNA replication and the cell cycle. Nutritional impoverishment prolongs the cell cycle, which further results in a decrease in *T*_rep_/T_cell_ despite an overall increase in *T*_rep_. As the duty cycle decreases, the enhancement effect of DNA replication may weaken, similar to the influence caused by solely altering Δ*θ*.

**FIGURE 5:**
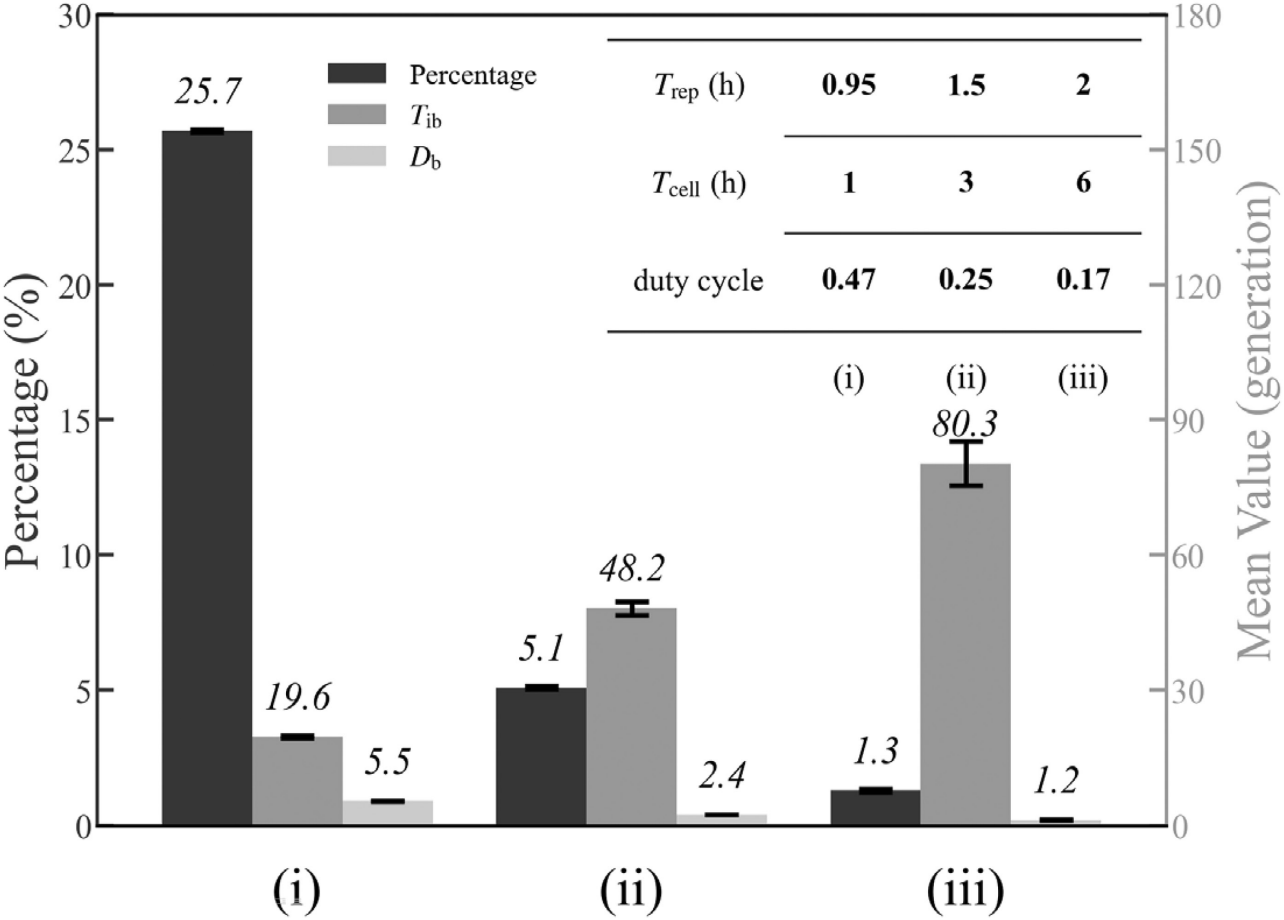
EPS production under varied cell cycle length with constant Spo0A‐P levels. The percentage of cells expressing EPS among a population and the averages of *T*_ib_ and *D*_b_ for different nutrient conditions. Δ*θ =* 84° and the input *S* (*t*) is set to 0.11 μM in all three cases. The inset presents the values of *T*_rep_, *T*_cell_, and the duty cycle of *g*_r_ pulsing. Error bars represent S.E.M.

When *T*_cell_ is set to 1 h, 3 h or 6 h, the corresponding *T*_rep_ and duty cycle of *g*_r_ pulsing are shown in the inset of Figure 5. We calculated the percentage (*P*) of EPS‐expressing cells (with *N*_EPS_ >10) in a colony of 600 cells. *P* is close to <*D*_b_>/<*T*_ib_> × 100%; *P* fluctuates around a mean value over time, and only its mean is presented for analysis purposes. *P* drops from 25.7% to 1.3% as *T*_cell_ rises from 1 h to 6 h (Figure 5). The decline in *P* is primarily driven by the reduced duty cycle of *g*_r_ pulsing, which leads to a remarkable increase in *T*_ib_ and a decrease in *D*_b_ (Figure 5). As shown in Figure 3F, the inhibition of target genes by SinR weakens during *g*_r_ = 2 periods. As the cell cycle extends, the *g*_r_ = 2 phase in each cycle shortens, decreasing its contribution to EPS production.

### 3.3 Complementary effects of Spo0A‐P and gr pulsing on EPS production

Given a constant Spo0A‐P level, the percentage of EPS‐expressing cells falls with diminishing nutrient availability in Figure 5, which does not align with the observation of its stable expression.^24^ Other mechanisms should be in place to facilitate EPS synthesis at low nutrient levels. It is known that Spo0A‐P indirectly promotes *eps* expression.^9^, ^41^ Possibly, the pulsing dynamics of Spo0A‐P compensate for the weakened promotion caused by the decreased duty cycle of *g*_r_.

It is established that the Spo0A‐P level undergoes oscillations under starvation due to a transient imbalance in *kinA* and *spo0F* gene dosage during replication and the negative feedback between KinA and Spo0F.^13^, ^14^ The angular difference (Δ*θ’*) between *kinA* and *spo0F* and *T*_rep_/*T*_cell_ determine the amplitude of Spo0A‐P oscillations (Figure S6); larger Δ*θ’* and smaller *T*_rep_/*T*_cell_ values lead to a larger amplitude.

**FIGURE 6:**
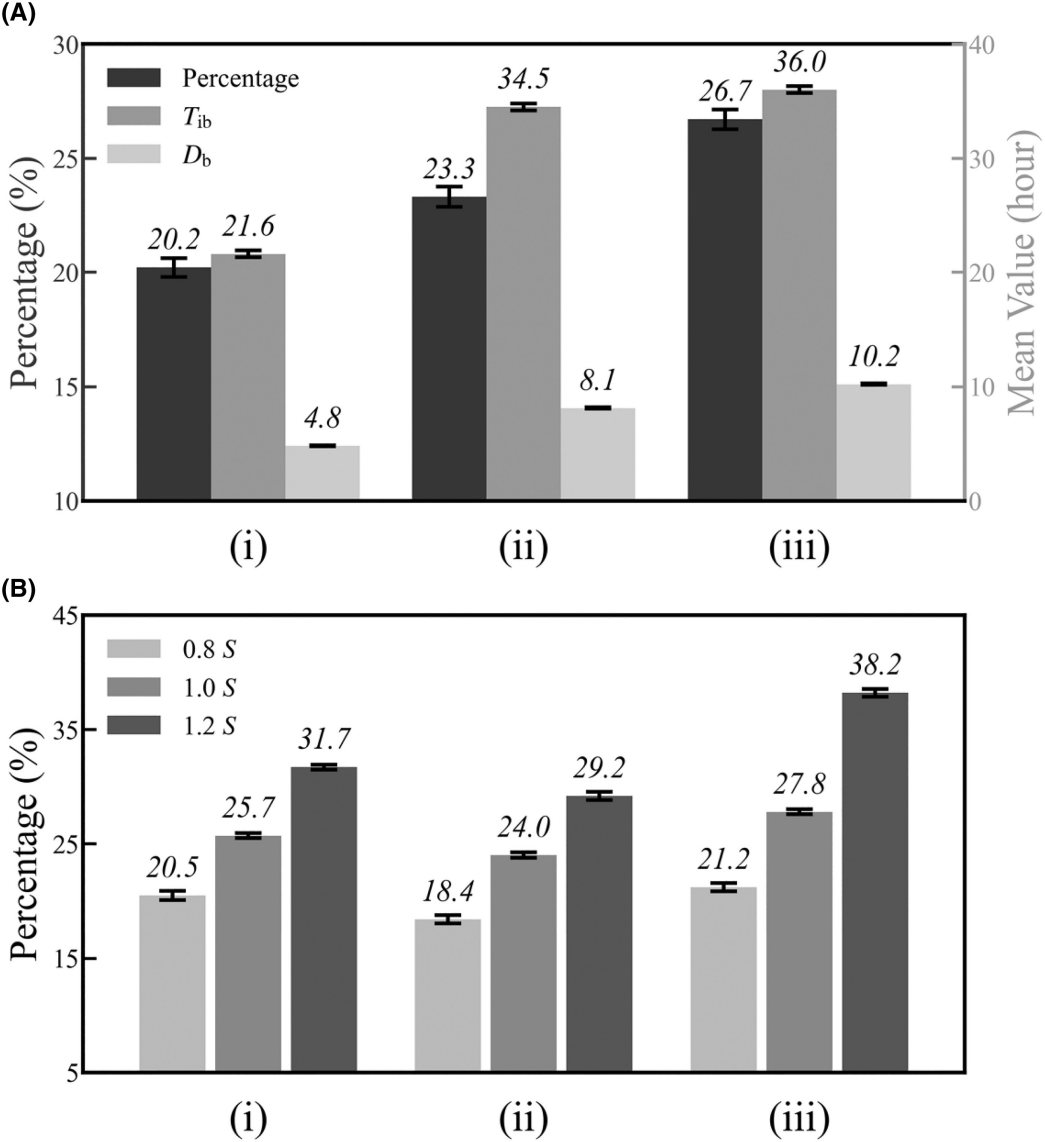
Complementary effects of Spo0A‐P oscillation and DNA replication engender stable EPS expression. The percentage of EPS‐secreting cells, *T*_ib_ and *D*_b_ under diverse nutrient conditions for Δ*θ* = 0° (A) and Δ*θ* = 84° (B). *S* (*t*) takes three forms as shown in Figure 2A, with *T*_cell_ set to 1 (i), 3 (ii) or 6 h (iii). In panel B, *S* (*t*) is also amplified by a factor of either 0.8 or 1.2. Error bars represent S.E.M.

For *T*_cell_ >1 h, *S*(*t*) oscillates periodically with the amplitude increasing with *T*_cell_ (Figure 2A). To elucidate how this impacts EPS production, suppose that *slrR* and *sinI‐sinR* are positioned adjacently on the chromosome (Δ*θ* = 0°). This configuration is unfavourable for DNA replication‐driven EPS production since *g*_r_ is fixed at 1 (Figure 3). Here, the enhanced EPS synthesis is primarily driven by higher Spo0A‐P (Figure 6A); the increase in *S*(*t*) leads to a larger *r* and a decrease in free SinR molecules. Meanwhile, *T*_ib_ and *D*_b_ rise with longer cell cycles, and the overall effect is to improve *P*.

At ∆*θ* = 84°, however, *P* stabilizes around 25% when *S*(*t*) has its default amplitude (Figure 6B), agreeing well with the experimental data.^24^ This stability results from the interplay between three factors: the duty cycle of *g*_r_ pulsing, proportion of the time when *m* remains high (i.e. *m* > *K*_IR_; *p*_m_), and cell cycle length. For short cell cycles, *S* remains low, and DNA replication dominates EPS production. As the cell cycle extends, the decreased duty cycle of *g*_r_ pulsing leads to a gradual reduction in its contribution to EPS production, but concurrent increases in Spo0A‐P abundance and *p*_m_ can compensate (Figure S6C). Furthermore, the Spo0A‐P level rises prominently upon completion of replication (Figure 2A), and its major contribution to EPS production primarily occurs later in each cycle. Consequently, stable EPS expression is maintained across diverse nutrient conditions. When the amplitude of *S*(*t*) is set to 1.2 times its default value, however, *p*_m_ rises evidently with increasing *T*_cell_ beyond 3 h while the duty cycle of *g*_r_ varies slightly (Figure S6C), resulting in a prominent increase in *P* (Figure 6B). These results indicate that *P* adapts to changes in *S*(*t*) amplitude, which awaits experimental justification.

Given that the core mechanism of the model relies on the antagonistic protein interactions, it is crucial to explore how the rate constants (*k*_IR_ and *k*_RrR_) governing SinR:SinI and SinR:SlrR formation affect *T*_ib_, *D*_b_ and *P*. Notably, the physiological values for *k*_IR_ and *k*_RrR_ can be experimentally determined,^17^ typically around 300 h^−1^ and 60 h^−1^ respectively (used as model defaults). Noise in SinR and SinI/SlrR production translates into fluctuating levels of free SinR through complex formation. While production and decay processes dictate the durations of high and low free SinR, the transient switching between these states occurs rapidly at physiologically relevant *k*_IR_ and *k*_RrR_ values. Consequently, variations in *k*_IR_ and *k*_RrR_ minimally influence the dwell times of free SinR in either state, rendering the average free SinR independent of *k*_IR_ and *k*_RrR_ (Figure S7). As SinR is a potent repressor, its target genes alternate between being expressed constantly and being silenced, and thus the durations of high and low free SinR directly determine EPS expression. As a result, changes to *k*_IR_ and *k*_RrR_ have minimal impact on the average levels of *T*_ib_, *D*_b_ and *P* (Figure S8).

## 4 DISCUSSION

Our model centers on a gene switch controlling EPS expression. This switch was once thought to be governed by the SinR‐SlrR PFL and driven by Spo0A‐P pulses.^1^ However, recent evidence reveals a novel mechanism involving SinR‐SinI antagonism, independent of the PFL.^22^ But the SinI‐SinR module alone cannot account for EPS secretion in 25% cells under nutrient scarcity.^24^ Here, we explored the roles of Spo0A‐P regulation and gene copy number variation. Spo0A‐P emerges as a crucial regulator, modulating the SinI/SinR synthesis rate ratio^23^ and influenced by multiple factors including DNA replication. The distant chromosomal locations of *slrR* and *sinI‐sinR* create transient copy number imbalance during replication, contributing to EPS expression. As nutrient deficiency extends the cell cycle, the duty cycle of *g*_r_ pulsing decreases while the Spo0A‐P pulsing amplitude increases, maintaining consistent EPS synthesis. Integrating these intricate mechanisms, our work offers a comprehensive understanding of switch activation and EPS production.

Nutrient gradients within a biofilm trigger bacterial cells to differentiate into diverse phenotypic states, enabling a form of division of labor that optimizes survival. The matrix production switch in *B. subtilis* can be activated by both external stimuli and internal fluctuations (intrinsic noise). The transcription of genes associated with matrix production (the *epsA‐O* and *tapA‐sipW‐tasA* operons) is directly repressed by SinR. Lord et al. demonstrated that a stochastic competition between SinI and SinR can trigger the switch even in the absence of SlrR.^22^ The total amounts of SinI and SinR modulate the switching frequency, while the SinR‐SlrR PFL extends the duration of matrix production once the switch is activated. Furthermore, Chen et al. combined theoretical modelling and experiments to explore how the cellular growth rate influences the distribution of matrix production proteins (e.g. TapA) within a bacterial population.^7^, ^42^ Their work reveals a complex interplay: A decrease in growth rate elevates the SinI/SinR ratio by increasing Spo0A‐P levels, whereas it lowers the SlrR/SinR ratio by reducing both the gene copy number ratio of *slrR* to *sinR* and the dilution rate. This results in an initial rise followed by a decrease in the average level of matrix production. Fluctuations in growth rate and intrinsic noise, amplified by the SinR‐SlrR PFL, are key factors driving the heterogeneity of matrix production across bacteria. On a single‐cell level, however, gene dosage and Spo0A‐P levels exhibit the cell‐cycle‐dependent pulsing behaviours.^13^ This introduces another layer of noise, likely contributing to the variability in matrix gene expression among cells. Our work highlights the importance of considering these factors. We showed that changes in gene dosage and Spo0A‐P levels throughout the cell cycle, influenced by both chromosomal arrangement and growth rate, play a crucial role in determining the proportion of cells actively producing biofilm matrix.

We propose that the strategy of bacteria responding to environment stress may be encoded through the chromosomal arrangement of pivotal genes like *kinA*, *spo0F*, *sinI‐sinR* and *slrR*. First, the *g*_r_ pulsing pattern mirrors the locations of *sinI‐sinR* and *slrR*. If ∆*θ* and *g*_r_ are altered by manipulating *sinI*‐*sinR* cassettes at different positions, distinct biofilm patterns appear, or biofilm formation is totally prevented^9^ (see Figure S9). Only around ∆*θ* = 90° does the fraction of EPS‐secreting cells remain consistent across diverse nutrient conditions (Figure S10), suggesting a crucial role for this specific arrangement. Second, Spo0A‐P dynamics also carry gene position information. Its pulsing behaviour depends on the positions of *kinA* and *spo0F*, with the pulse height and width determined by their angular distance (Δ*θ’*). Small Δ*θ’* disrupts pulsing, leading to low Spo0A‐P levels.^14^ Third, analysis of 28 *B. subtilis* strains reveals remarkably consistent ∆*θ* values, with few exceptions (Table S3). Similarly, Δ*θ’* shows small variation across 45 *B. subtilis* species.^14^ These observations strongly suggest that gene positions, together with the resulting transient imbalances in copy number during DNA replication, are used to encode expression patterns. Therefore, our work unveils a novel mechanism for EPS regulation in *B. subtilis*, where chromosomal location of key genes encodes information for stable EPS production through *g*_r_ pulsing and Spo0A‐P dynamics. This mechanism appears evolutionarily conserved.

Biofilms, with their enhanced nutrient uptake efficiency, offer a distinct advantage for bacteria under various environmental conditions. Within a colony, some cells secrete EPS, which promotes biofilm formation by effectively capturing neighbouring cells.^24^ By keeping a constant proportion of EPS‐producing cells within the colony, biofilm nucleation will be triggered once the cell density reaches a certain threshold under any condition. An appropriate proportion is required: excessive EPS production (at high proportion) can force colonies to adhere to surfaces, limiting their mobility; at low proportion, biofilms may not form properly. The mechanism proposed here is both accurate and flexible; the proportion can vary with the Spo0A‐P pulsing amplitude (Figure 6B). Overall, our results suggest that biofilm formation in *B. subtilis* may be controlled by an internal ‘timer’, rather than solely relying on external environmental cues.

While our focus was on individual cells, the collective behaviours in a colony, such as quorum sensing, also play a crucial role in biofilm formation. Integrating this aspect into the model will offer a more holistic understanding. Probing the role for stable EPS expression across different environmental conditions will further elucidate its contribution to biofilm robustness and adaptability.

## 5 CONCLUSIONS

The current work reveals how chromosomal arrangement of key genes couples DNA replication with the biofilm gene switch activation. We verified that EPS production occurs in bursts. Both high levels of Spo0A‐P and a large duty cycle of *g*_r_ pulsing facilitate EPS production. Since *g*_r_ = 2 and high Spo0A‐P levels appear in distinct phases of the cell cycle, together with the decreased duty cycle and increased Spo0A‐P amplitude with extending the cell cycle, stable EPS synthesis within colonies can be sustained under varying nutrient conditions. Our results shed new light on the regulation of EPS expression and biofilm formation in *B. subtilis*.

## AUTHOR CONTRIBUTIONS

**Renjie Wu:** Conceptualization (lead); data curation (lead); formal analysis (lead); investigation (lead); writing – original draft (equal); writing – review and editing (lead). **Ling‐Xing Kong:** Data curation (lead); formal analysis (lead); investigation (lead); writing – original draft (lead); writing – review and editing (equal). **Feng Liu:** Conceptualization (lead); funding acquisition (lead); investigation (equal); project administration (lead); resources (lead); supervision (lead); writing – original draft (lead); writing – review and editing (lead).

## FUNDING INFORMATION

This work was supported by the National Natural Science Foundation of China (11874209).

## CONFLICT OF INTEREST STATEMENT

The authors confirm that there are no conflicts of interest.

## Supporting information


Data S1.

## Data Availability

All data needed to evaluate the conclusions in the paper are present in the paper. Custom python codes for reproducing the simulation and analysis are available at https://github.com/Biophysics‐nju.
