# Supplementary material for: Regulation of biofilm gene expression by DNA replication in Bacillus subtilis
Source: J Cell Mol Med. 2024 Jun 20;28(12):e18481. doi: 10.1111/jcmm.18481 (PMC11187747; doi:10.1111/jcmm.18481)
Supplement: Supplementary file 1 — Data S1. [file JCMM-28-e18481-s001.pdf]

Supplemental Information

Regulation of biofilm gene expression by DNA replication in *Bacillus subtilis*

Renjie Wu, Ling-Xing Kong and Feng Liu

This document includes the model for Spo0A-P pulsing, 10 figures and 4 tables.

Text: Mathematical model for Spo0A-P pulsing

We used a previous mathematical model of the sporulation phosphorelay network to characterize the pulsatile dynamics of phosphorylated spo0A (1-2). The dynamics of the network can be described by the following ordinary differential equations (ODEs).

$$\frac{d[\mathrm{K_t}]}{dt} = \frac{g_{kinA}}{F(\mu)}\left(v_{kinA}^{0} + v_{kinA}^{\max}\frac{m^n}{K_{kinA}+m^n}\right) - (k_d+\mu)[\mathrm{K_t}] \quad (1)$$

$$\frac{d[\mathrm{F_t}]}{dt} = \frac{g_{0F}}{F(\mu)}\left(v_{0F}^{0} + v_{0F}^{\max}\frac{m^n}{K_{0F}+m^n}\right) - (k_d+\mu)[\mathrm{F_t}] \quad (2)$$

$$\frac{d[\mathrm{B_t}]}{dt} = \frac{g_{0B}}{F(\mu)}v_{0B} - (k_d+\mu)[\mathrm{B_t}] \quad (3)$$

$$\frac{d[\mathrm{A_t}]}{dt} = \frac{g_{0A}}{F(\mu)}\left(v_{0A}^{0} + v_{0A}^{\max}\frac{m^n}{K_{0A}+m^n}\right) - (k_d+\mu)[\mathrm{A_t}] \quad (4)$$

$$\frac{d[\mathrm{E_t}]}{dt} = v_{0E} - (k_d+\mu)[\mathrm{E_t}] \quad (5)$$

$$\frac{d[\mathrm{R_t}]}{dt} = v_{Rap} - (k_d+\mu)[\mathrm{R_t}] \quad (6)$$

$$\frac{d[\mathrm{K_P}]}{dt} = k_\mathrm{p}[\mathrm{K}] - k_\mathrm{dp}[\mathrm{K_p}] - k_\mathrm{b}[\mathrm{K_p}][\mathrm{F}] + k_1[\mathrm{K_pF}] - (k_d+\mu)[\mathrm{K_P}] \quad (7)$$

$$\frac{d[\mathrm{K_pF}]}{dt} = k_\mathrm{b}[\mathrm{K_p}][\mathrm{F}] + k_\mathrm{b}[\mathrm{K}][\mathrm{F_p}] - (k_1+k_2)[\mathrm{K_pF}] - (k_d+\mu)[\mathrm{K_pF}] \quad (8)$$

$$\frac{d[\mathrm{F_p}]}{dt} = -k_\mathrm{b}[\mathrm{F_p}]([\mathrm{K}]+[\mathrm{R}]+[\mathrm{B}]) + k_2[\mathrm{K_pF}] + k_4[\mathrm{F_pB}] + \mathrm{k_8}[\mathrm{F_pR}] - \mathrm{k_{dpa}}[\mathrm{F_p}] - (k_d+\mu)[\mathrm{F_p}] \quad (9)$$

$$\frac{d[\mathrm{KF}]}{dt} = k_\mathrm{b}[\mathrm{K}][\mathrm{F}] - k_3[\mathrm{KF}] - (k_d+\mu)[\mathrm{KF}] \quad (10)$$

$$\frac{d[\mathrm{B_p}]}{dt} = -k_\mathrm{b}[\mathrm{B_p}]([\mathrm{F}]+[\mathrm{A}]) + k_5[\mathrm{F_pB}] + k_6[\mathrm{B_pA}] - (k_d+\mu)[\mathrm{B_p}] \quad (11)$$

$$\frac{d[\mathrm{F_pB}]}{dt} = k_\mathrm{b}([\mathrm{F_p}][\mathrm{B}] + [\mathrm{F}][\mathrm{B_p}]) - (k_4 + k_5)[\mathrm{F_pB}] - (k_d + \mu)[\mathrm{F_pB}] \tag{12}$$

$$\frac{d[\mathrm{A_p}]}{dt} = -k_\mathrm{b}[\mathrm{A_p}]([\mathrm{B}] + [\mathrm{E}]) + k_7[\mathrm{B_pA}] + k_{10}[\mathrm{A_pE}] - \mathrm{k_{dpa}}[\mathrm{A_p}] + \mathrm{k_{pa}}[\mathrm{A}] - (k_d + \mu)[\mathrm{A_p}] \tag{13}$$

$$\frac{d[\mathrm{B_pA}]}{dt} = k_\mathrm{b}([\mathrm{B_p}][\mathrm{A}] + [\mathrm{B}][\mathrm{A_p}]) - (k_6 + k_7)[\mathrm{B_pA}] - (k_d + \mu)[\mathrm{B_pA}] \tag{14}$$

$$\frac{d[\mathrm{F_pR}]}{dt} = k_\mathrm{b}[\mathrm{F_p}][\mathrm{R}] - (\mathrm{k_8} + \mathrm{k_9})[\mathrm{F_pR}] - (k_d + \mu)[\mathrm{F_pR}] \tag{15}$$

$$\frac{d[\mathrm{A_pE}]}{dt} = k_\mathrm{b}[\mathrm{A_p}][\mathrm{E}] - (k_{10} + k_{11})[\mathrm{A_pE}] - (k_d + \mu)[\mathrm{A_pE}] \tag{16}$$

$$\frac{dm}{dt} = (k_s + \mu)([\mathrm{A_p}] - m) \tag{17}$$

$$[\mathrm{K}] = [\mathrm{K_t}] - ([\mathrm{K_p}] + [\mathrm{K_pF}] + [\mathrm{KF}]) \tag{18}$$

$$[\mathrm{F}] = [\mathrm{F_t}] - ([\mathrm{F_p}] + [\mathrm{K_pF}] + [\mathrm{KF}] + [\mathrm{F_pB}] + [\mathrm{F_pR}]) \tag{19}$$

$$[\mathrm{B}] = [\mathrm{B_t}] - ([\mathrm{B_p}] + [\mathrm{F_pB}] + [\mathrm{B_pA}]) \tag{20}$$

$$[\mathrm{A}] = [\mathrm{A_t}] - ([\mathrm{A_p}] + [\mathrm{B_pA}] + [\mathrm{A_pE}]) \tag{21}$$

$$[\mathrm{R}] = [\mathrm{R_t}] - [\mathrm{F_pR}] \tag{22}$$

$$[\mathrm{E}] = [\mathrm{E_t}] - [\mathrm{A_pE}] \tag{23}$$

K, F, B, A, R and E denote the proteins KinA, Spo0F, Spo0B, Spo0A and Spo0E, respectively. Subscript p marks the phosphorylated form of proteins, and subscript t marks the total amount of proteins. $\mathrm{A_pF}$, AF, $\mathrm{F_pB}$, $\mathrm{B_pA}$, $\mathrm{F_pR}$, $\mathrm{A_pE}$ refer to Spo0$\mathrm{A_p}$:Spo0F, Spo0A:Spo0F, Spo0$\mathrm{F_p}$:Spo0B, Spo0$\mathrm{B_p}$:Spo0A, Spo0$\mathrm{F_p}$:Rap and Spo0$\mathrm{A_p}$:Spo0E, respectively, with colon denoting protein complexes. The parameter values and their descriptions are shown in Table S4.

In this model, the copy number of *kinA* is $g_\mathrm{kinA} = 2$ because the *kinA* gene is proximal to the chromosome origin, while g_i (i = *kinA*, *spo0A*, *spo0B*) equals 1 during DNA replication and 2 after DNA replication, i.e.,

$$g_\mathrm{i}(t) = \begin{cases} 1 & (t \bmod T_\mathrm{cell}) < T_\mathrm{rep} \\ 2 & (t \bmod T_\mathrm{cell}) \geq T_\mathrm{rep} \end{cases},$$

where the cell cycle duration (T_cell) and DNA replication period (T_rep) depend on the

cell growth rate (μ). $T_{\text{cell}} = \log 2/\mu$ h and $T_{\text{rep}} = 0.78+0.15/\mu$ h. $F(\mu)$ is a proportionality factor: $F(\mu) = a\mathrm{e}^{b\mu}+c$, with $a = 0.690$, $b = 0.689$ and $c = 0.745$.

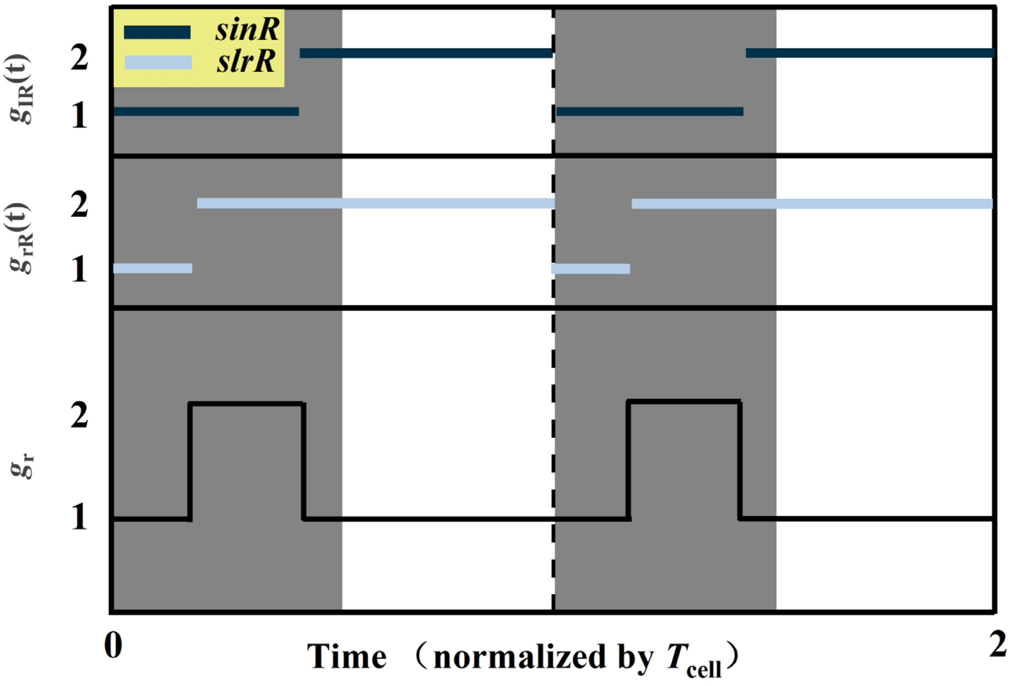

Figure S1. Temporal evolution of gene copy numbers in individual cells. Time courses of g_{IR}, g_{rR} and g_r (= g_{rR}/g_{IR}) (from top to bottom)**.** The durations of the cell cycle and replication period are 3 h and 1.5 h, respectively.

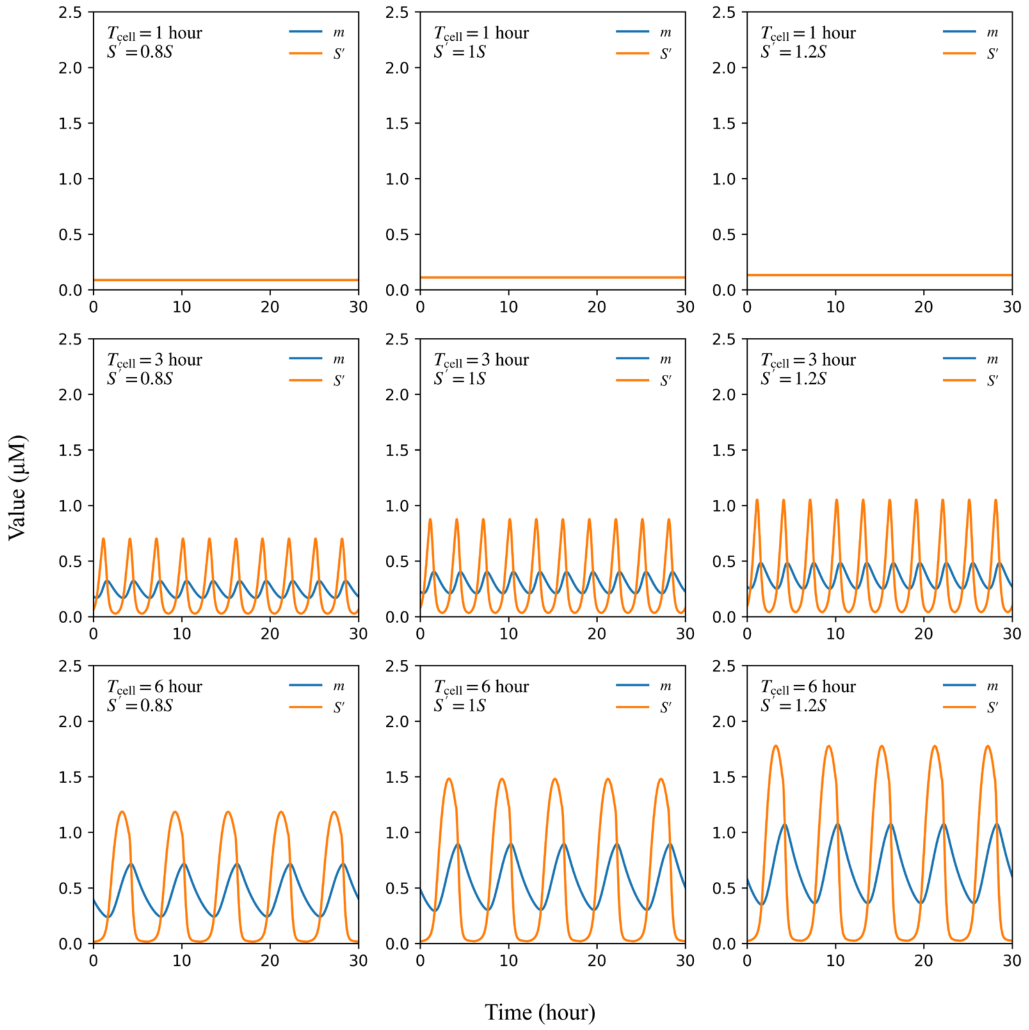

Figure S2. Dynamics of the Spo0A-P level (S) and intermediate variable (m). The cell cycle period (T_{cell}) is set to 1, 3 or 6 h. The parameter values are also changed to make the amplitude of $S(t)$ equal 0.8 or 1.2 times its default value (to differentiate between these cases, the Spo0A-P level is denoted by S') . Of note, S and m are almost identical for $T_{cell} = 1$ h.

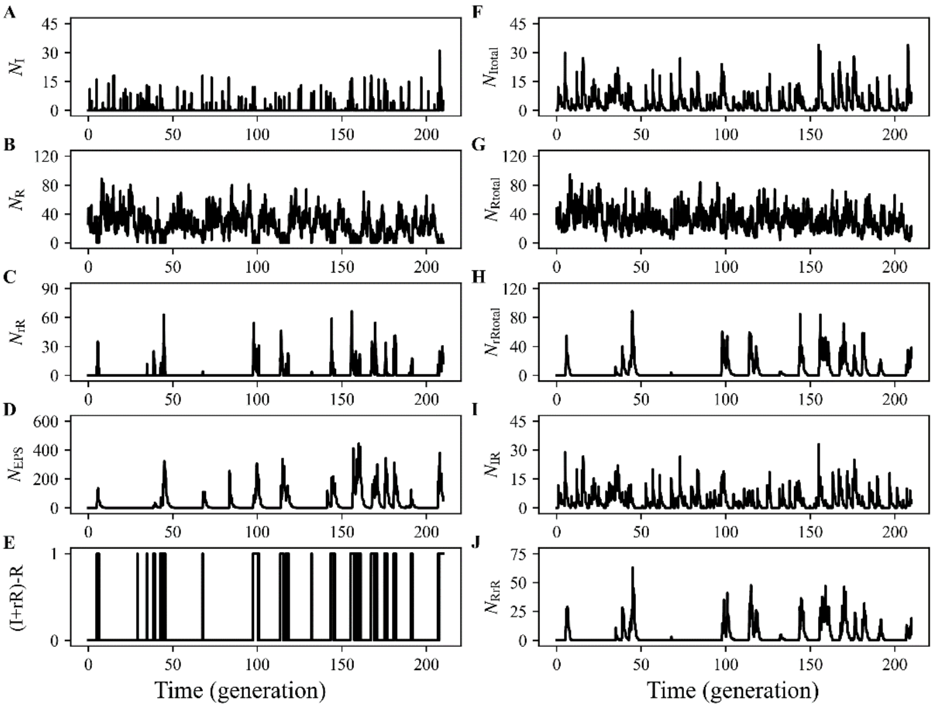

Figure S3. Time courses of the numbers of network components on a single simulation trial. The label '(I+rR)-R' quantifies the relationship in size between the total amount of both SinI and SlrR and that of SinR, where a magnitude of 1 signifies a predominance of the former, and vice versa. T_{cell} = 1 h.

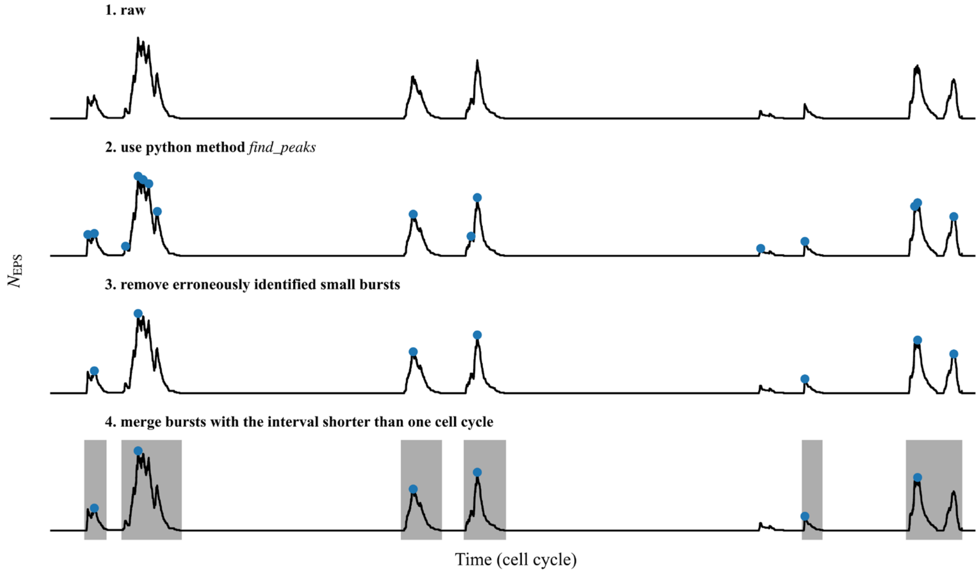

Figure S4. Process of burst identification. From top to bottom: raw data, initial burst data obtained using the *find_peaks* method, data after removal of erroneously identified small bursts, and data after merging bursts with the interval shorter than one cell cycle. The reported statistical data are extracted from the bottom graph.

The statistical results on inter-burst interval (T_{ib}) and burst duration (D_b) in the main text were obtained using a consistent calculation approach. Initially, we applied a smoothing process to the time series. We then utilized the *find_peaks* function from the signal processing submodule of the built-in scientific computing library *scipy* in Python. This method enables the identification of burst positions and burst widths. We further eliminated minor bursts falsely recognized by this method. Owing to protein dilution, we could not tell whether the number of molecules decreased between bursts of reporter fluorescence intensity occurring within a time window shorter than one cell cycle. Thus, we merged bursts occurring within a window shorter than one cell cycle. Ultimately, we acquired the resulting burst data.

When constructing histograms for noise analysis, we excluded all bursts with heights shorter than fluctuations in the background signal, i.e., the number of EPS proteins within a burst should exceed a threshold N_{th} (e.g., 10). Notably, the statistics of the burst duration, burst interval and proportion of EPS-expressing cells are insensitive to the concrete value of N_{th} provided it is small (see Figure S5).

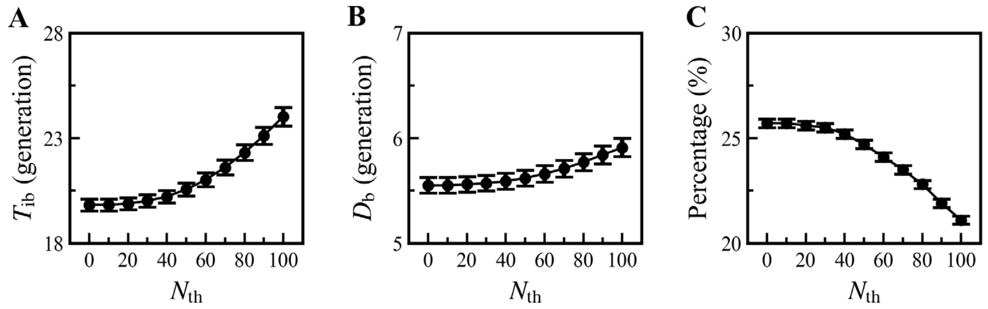

Figure S5. Burst statistics according to different baseline thresholds. The boundaries of EPS bursts are defined as the points where N_{EPS} crossed a specified 'background' threshold (N_{th}). (A) The mean burst interval (T_{ib}) and (B) burst duration (D_b) and (C) the percentage (P) of EPS-expressing cells are influenced by the value of N_{th}. Because $1/T_{ib}$, $1/D_b$ and P are low-pass filtered relative to N_{th}, the quantities above remain unchanged when $N_{th} < 20$. Here, $T_{cell} = 1$ h.

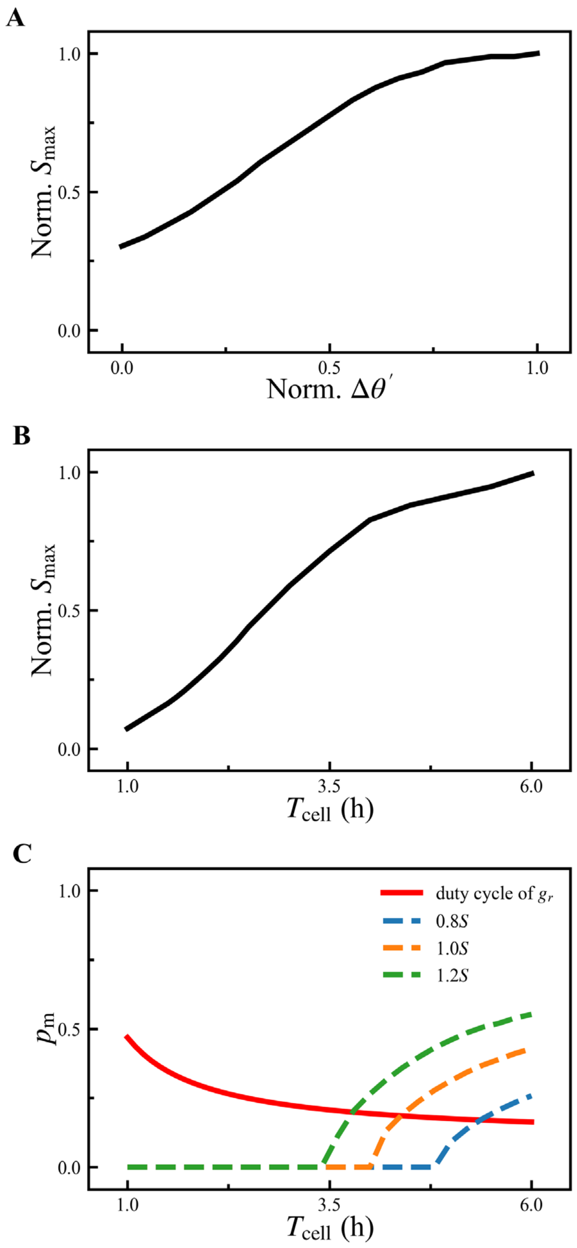

Figure S6. Variations in Spo0A-P dynamics under diverse conditions. Changing the angular difference ($\Delta\theta'$) between *kinA* and *spo0F* (A) or the cell cycle length (B) alters the amplitude of Spo0A-P oscillation. (C) The proportion (p_m) of the time when m remains high (i.e., $m > K_{IR}$) when the amplitude of $S(t)$ is set to 0.8, 1, or 1.2 times its default value. The red solid line shows the duty cycle of g_r pulsing versus T_{cell} when the amplitude of $S(t)$ has its default value.

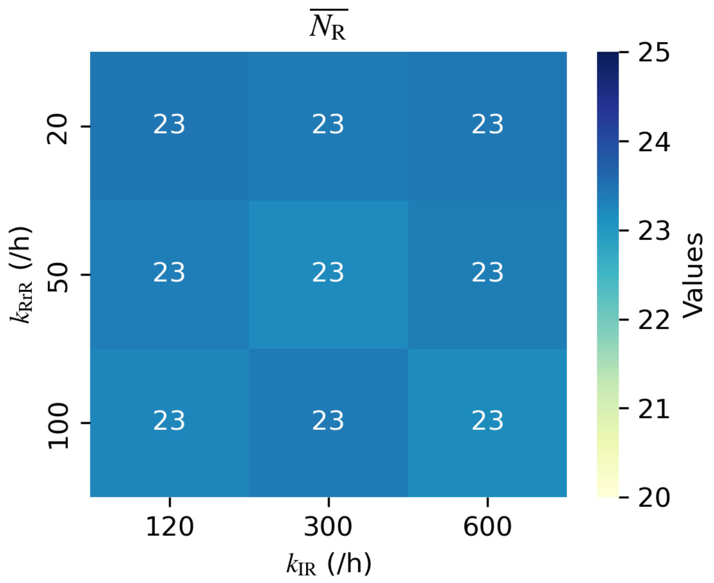

Figure S7. Heatmap for the average number of free SinR as functions of the complex formation rate constants. At $T_{cell} = 1$ h, the variations in the rate constant of SinI:SinR formation ($k_{IR} = 120, 300, 600$ h^{-1}) and that of SinR:SlrR formation ($k_{RrR} = 20, 50, 100$ h^{-1}) have minor impact on the time-averaged number of free SinR (N_R).

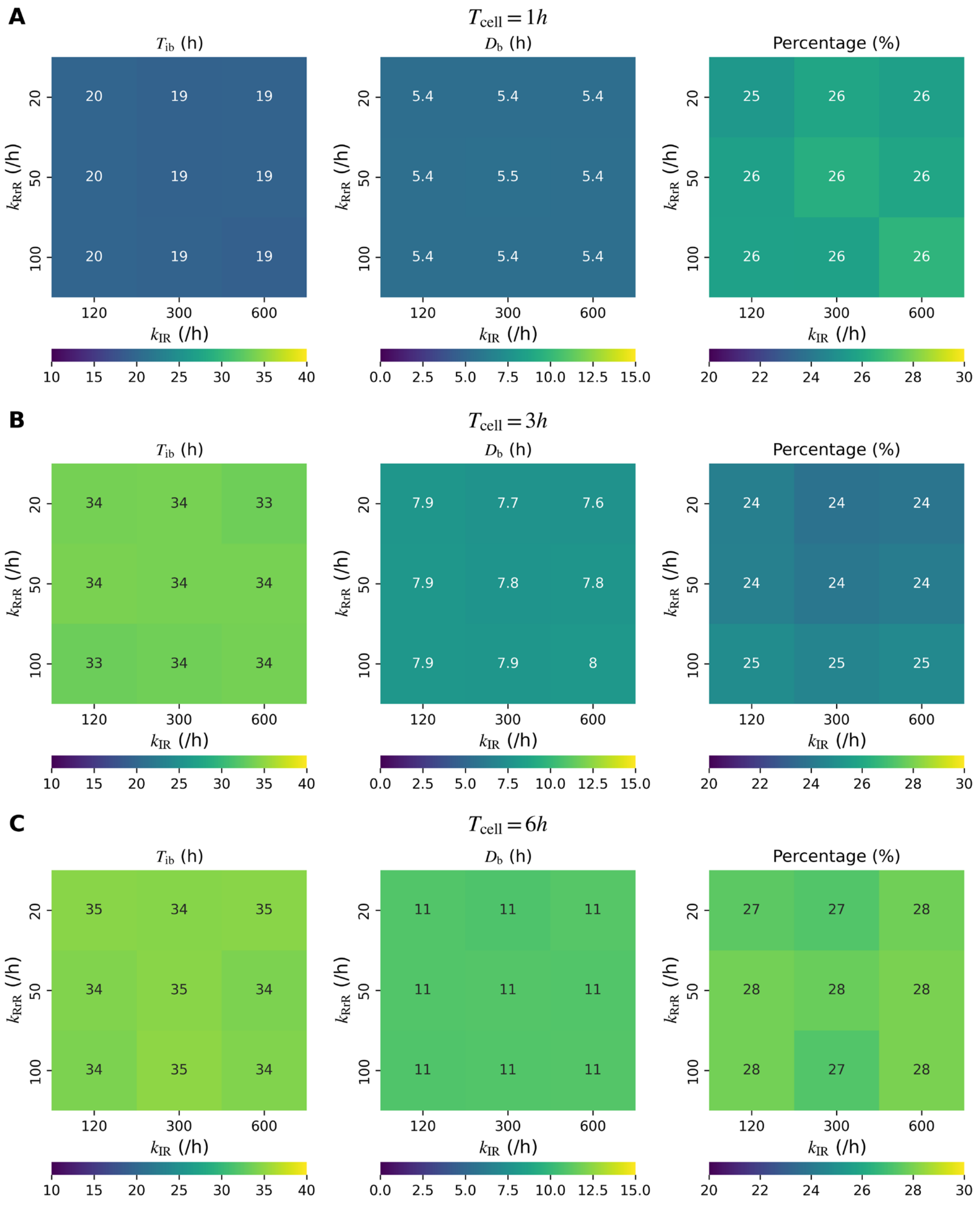

Figure S8. Impact of the rate constants of complex formation on T_{ib}, D_b and P. Shown are the heatmaps for T_{ib}, D_b and P (from left to right) as functions of the rate constants of SinI:SinR formation (k_{IR}) and SinR:SlrR formation (k_{RrR}) for T_{cell} = 1 h (A), 3 h (B) or 6 h (C). k_{IR} is greater than k_{RrR}, ensuring that SinR has a higher binding affinity for SinI than SlrR. Overall, changes to k_{IR} and k_{RrR} have minor effects on T_{ib}, D_b and P.

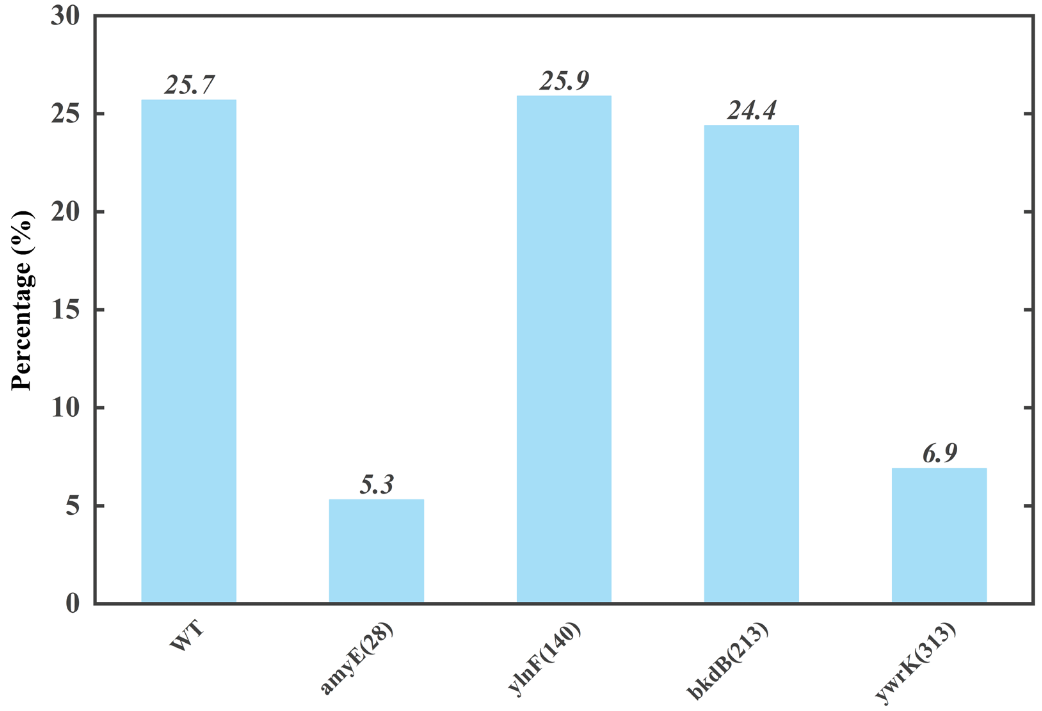

Figure S9. Percentages of EPS-expressing cells in a colony under different conditions.

Chai *et al*. (3) inserted the sinI-sinR cassette into sinI-sinR mutant strains and found that the effectiveness of functional recovery depended on the choice of insertion site. They inserted the sinI-sinR cassette at five different sites; three insertion sites near the Ter site could support biofilm formation, in contrast to two insertion sites near the OriC site.

To mimic the experimental protocol, we adjusted g_{IR}. Experimentally, the *slrR* gene is located at 302°, and *sinI-sinR* is placed at the corresponding locations shown in the figure. While keeping $g_{rR}(t)$ fixed, we changed the t_r of $g_{IR}(t)$ and performed simulation with T_{cell} = 3 h. The results are in agreement with the experimental data, indicating that insertion sites near OriC are ineffective in biofilm synthesis due to their close proximity to the *slrR* gene.

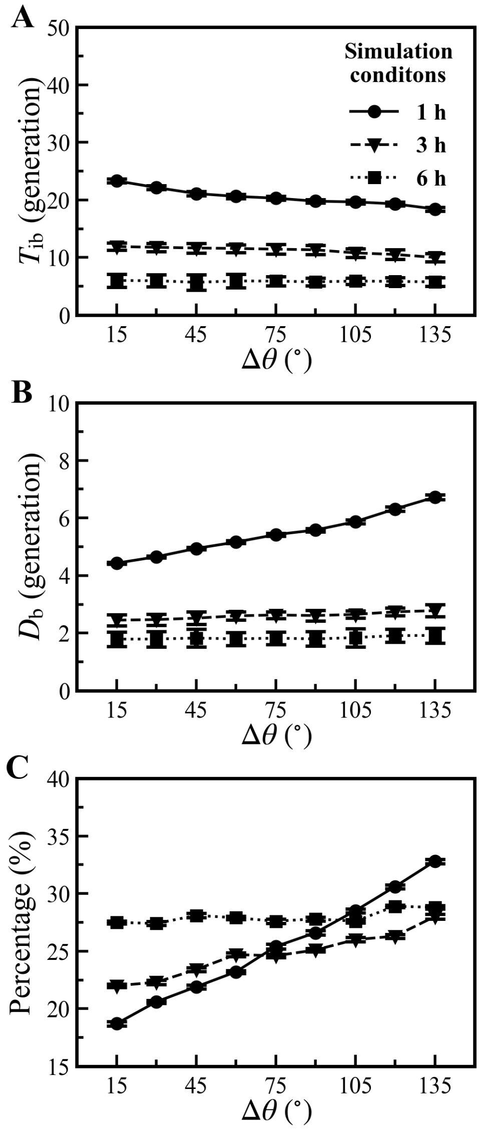

Figure S10. Impact of chromosomal arrangement of genes on EPS dynamics under diverse conditions. Mean values of T_{ib} (*A*) and D_b (*B*) and the percentage of EPS-expressing cells (*C*) versus the angular difference ($\Delta\theta$) between *slrR* and *sinI-sinR*. T_{cell} = 1 h, 3 h, or 6 h, and the input $S(t)$ takes the same form as in Figure 6B.

Table S1. Parameters in the model

Parameters with μ = log2 h^{-1} and after division (i.e., the cell volume is equal to V_0).

Parameter	Name	Value	Reference
b_R	Average burst size of protein SinR	10	(4)
b_I	Average burst size of protein SinI	10	(4)
b_{rR}	Average burst size of protein SlrR	5	(5)
b_Z	Average burst size of protein Z	5	(5)
λ_R	Maximum transcription rate of *sinR* under single gene dosage	22/h	Fit to (6)
$\Delta\lambda_R$	Basal transcription rate of *sinR* under single gene dosage	5/h	(7)
λ_I	Maximum transcription rate of *sinI* under single gene dosage	22/h	Fit to (6)
$\Delta\lambda_I$	Basal transcription rate of *sinI* under single gene dosage	0	(3)
λ_{rR}	Maximum transcription rate of *slrR* under single gene dosage	5/h	estimated
λ_Z	Maximum transcription rate of Z under single gene dosage	5/h	estimated
n_R	Hill coefficient in SinR expression	2	estimated
n_I	Hill coefficient in SinI expression	2	estimated
n_{rR}	Hill coefficient in SlrR expression	4	(8)
n_Z	Hill coefficient in Z expression	4	(8)
K_R	Dissociation constant in SinR expression	0.6μM	estimated
K_I	Dissociation constant in SinI expression	0.6μM	estimated
K_{rR}	Dissociation constant in SlrR expression	5	estimated
K_Z	Dissociation constant in Z expression	5	estimated
d_R	Degradation rate constant of SinR	0.5/h	(7)
d_I	Degradation rate constant of SinI	0.5/h	(7)
d_{rR}	Degradation rate constant of SlrR	1/h	(7)
d_Z	Degradation rate constant of Z	0.5/h	estimated
k_{IR}	Dimerization rate of SinI:SinR	300	(9)
d_{IR}	Degradation rate constant of SinI:SinR	0.5/h	estimated
k_{RrR}	Dimerization rate of SlrR:SinR	60/h	(9)
d_{RrR}	Degradation rate constant of SlrR:SinR	0.5/h	estimated
k_s	Parameter for time delay caused by indirect controlled of Spo0A-P	0.3/h	(1)

Here Z represents EPS proteins.

Table S2. The update law for stochastic simulation

The simulation was performed using the Gillespie algorithm. The update law is given in the table below. The volume ratio (v) equals V/V_0.

Reactions	Rate	Update
$\emptyset \rightarrow n\mathrm{R}$	$\frac{g_{\mathrm{R}}(t)}{F(\mu)}\left[\lambda_R \frac{s(t)^{n_R}}{s(t)^{n_R}+K_R^{n_R}} + \Delta\lambda_{\mathrm{R}}\right] v$	$N_{\mathrm{R}} \rightarrow N_{\mathrm{R}} + n$ with probability $P(n) = \frac{b_R^n}{(b_R+1)^{n+1}}$
$\mathrm{R} \rightarrow \emptyset$	$d_{\mathrm{R}} N_R$	$N_{\mathrm{R}} \rightarrow N_{\mathrm{R}} - 1$
$\emptyset \rightarrow n\mathrm{I}$	$\frac{g_{\mathrm{I}}(t)}{F(\mu)}\left[\lambda_R \frac{s(t)^{n_I}}{s(t)^{n_I}+K_I^{n_I}} + \Delta\lambda_{\mathrm{I}}\right] v$	$N_{\mathrm{I}} \rightarrow N_{\mathrm{I}} + n$ with probability $P(n) = \frac{b_I^n}{(b_I+1)^{n+1}}$
$\mathrm{I} \rightarrow \emptyset$	$d_{\mathrm{I}} N_{\mathrm{I}}$	$N_{\mathrm{I}} \rightarrow N_{\mathrm{I}} - 1$
$\emptyset \rightarrow n\mathrm{rR}$	$\frac{g_{\mathrm{rR}}(t)}{F(\mu)} \lambda_{\mathrm{rR}} \frac{[R]^{n_{rR}}}{[R]^{n_{rR}}+K_{rR}^{n_{rR}}} v$	$N_{\mathrm{rR}} \rightarrow N_{\mathrm{rR}} + n$ with probability $P(n) = \frac{b_{rR}^n}{(b_{rR}+1)^{n+1}}$
$\mathrm{rR} \rightarrow \emptyset$	$d_{\mathrm{rR}} N_{\mathrm{rR}}$	$N_{\mathrm{rR}} \rightarrow N_{\mathrm{rR}} - 1$
$\emptyset \rightarrow n\mathrm{Z}$	$\frac{g_{\mathrm{Z}}(t)}{F(\mu)} \lambda_Z \frac{[R]^{n_Z}}{[R]^{n_Z}+K_Z^{n_Z}} v$	$N_{\mathrm{Z}} \rightarrow N_{\mathrm{Z}} + n$ with probability $P(n) = \frac{b_Z^n}{(b_Z+1)^{n+1}}$
$\mathrm{Z} \rightarrow \emptyset$	$d_{\mathrm{Z}} N_Z$	$N_Z \rightarrow N_{\mathrm{Z}} - 1$
$I + R \rightarrow IR$	$\frac{k_{\mathrm{IR}}}{v} N_{\mathrm{I}} N_R$	$N_{\mathrm{I}} \rightarrow N_{\mathrm{I}} - 1$; $N_{\mathrm{R}} \rightarrow N_{\mathrm{R}} - 1$; $N_{\mathrm{IR}} \rightarrow N_{\mathrm{IR}} + 1$
$IR \rightarrow \emptyset$	$d_{\mathrm{IR}} N_{IR}$	$N_{\mathrm{IR}} \rightarrow N_{\mathrm{IR}} - 1$
$rR + R \rightarrow RrR$	$\frac{k_{\mathrm{RrR}}}{v} N_{\mathrm{rR}} N_R$	$N_{\mathrm{rR}} \rightarrow N_{\mathrm{rR}} - 1$; $N_{\mathrm{R}} \rightarrow N_{\mathrm{R}} - 1$; $N_{\mathrm{RrR}} \rightarrow N_{\mathrm{RrR}} + 1$
$RrR \rightarrow \emptyset$	$d_{\mathrm{RrR}} N_{RrR}$	$N_{\mathrm{RrR}} \rightarrow N_{\mathrm{RrR}} - 1$

The number of proteins is halved per division.

Table S3. Genome length and chromosomal locations of *sinI-sinR* and *slrR* in 28 bacteria

The gene location is measured clockwise from the replication origin (0° = 360° = chromosome origin; 180° = chromosome terminus). Data were drawn from the PubMed Gene Database.

Species name	Genome length	*sinI-sinR* location (degrees/360)	*slrR* location (degrees/360)	Δ*θ* (degrees)
Bacillus subtilis subsp. subtilis str. 168	4215606	218.0	301.5	83.5
Bacillus amyloliquefaciens DSM 7	3980199	218.9	302.9	84.0
Bacillus subtilis strain NCIB 3610	4215607	218.0	301.5	83.5
Bacillus subtilis subsp. spizizenii str. W23	4027676	215.3	297.6	82.3
Bacillus licheniformis DSM 13 = ATCC 14580	4222645	217.4	300.4	83.0
Bacillus paralicheniformis ATCC 9945a	4376305	223.1	303.1	80.0
Bacillus subtilis subsp. subtilis 6051-HGW	4215610	218.0	301.5	83.5
Bacillus subtilis QB928	4146839	219.8	300.5	80.7
Bacillus amyloliquefaciens subsp. plantarum YAU B9601-Y2	4242774	226.2	301.5	75.4
Bacillus amyloliquefaciens TA208	3937511	221.3	303.2	81.9
Bacillus amyloliquefaciens subsp. plantarum CAU B946	4019861	225.5	304.6	79.1
Bacillus amyloliquefaciens subsp. plantarum UCMB5036	3910324	222.5	300.8	78.3
Bacillus amyloliquefaciens subsp. plantarum UCMB5113	3889532	223.1	303.0	79.9
Bacillus amyloliquefaciens subsp. plantarum NAU-B3	4196170	115.7	301.8	186.1 (57.5)
Bacillus spizizenii ATCC 6633 = JCM 2499 strain ATCC 6633	4045538	215.9	297.9	82.0
Bacillus vallismortis strain BL01	4115091	218.2	296.5	78.3

Bacillus inaquosorum strain LBA001	4200707	50.9	151.8	100.8
Bacillus inaquosorum strain BSXE-2102	4242131	214.4	298.5	84.2
Bacillus inaquosorum strain 1HC-NA	4240317	154.1	70.0	84.2
Bacillus halotolerans strain MEC_B334	4280526	219.4	299.3	79.9
Bacillus halotolerans strain Q2H2	4155130	215.3	296.7	81.4
Bacillus halotolerans strain KF17	4151079	215.2	296.6	81.4
Bacillus stercoris strain BS21	4780609	237.0	308.3	71.2
Bacillus subtilis BEST7613	7585470	197.9	327.5	129.6
Bacillus amyloliquefaciens CC178	3916828	222.7	301.9	79.2
Bacillus halotolerans strain ZB201702	4154245	321.0	234.9	-86.1
Bacillus stercoris BST19	4167147	8.3	282.3	274.0 (-69.4)
Bacillus stercoris strain SMPL712	4136111	15.2	292.1	277.0 (-52.7)

$\Delta\theta$ with parentheses denotes the effective $\Delta\theta$, which can be used to calculate t_r. When two genes are positioned on the left and right sides of the chromosomal ring (Figure 1 C), their effective $\Delta\theta$ is not directly obtained by subtracting them. Instead, it is calculated by symmetrizing the two genes to the same side.

Table S4. Parameter values in the model for Spo0A-P pulsing

Parameter	Description	Value
k_{p}	KinA auto-phosphorylation	12 h^{-1}
k_{dp}	KinA dephosphorylation	1 h^{-1}
k_{b}	Diffusion-limited binding rate constant	5000 $\mu M^{-1}h^{-1}$
k_1	KinA$_{\mathrm{P}}$:Spo0F complex dissociation	500 h^{-1}
k_2	Spo0F phosphorylation	300 h^{-1}
k_3	KinA:Spo0F complex dissociation	5000 h^{-1}
k_4	Spo0F$_{\mathrm{P}}$:Spo0B complex dissociation	200 h^{-1}
k_5	Spo0F to Spo0B phosphotransfer	800 h^{-1}
k_6	Spo0B$_{\mathrm{P}}$:Spo0A complex dissociation	200 h^{-1}
k_7	Spo0B to Spo0A phosphotransfer	800 h^{-1}
k_8	Spo0F$_{\mathrm{P}}$:Rap complex dissociation	100 h^{-1}
k_9	Spo0F$_{\mathrm{P}}$ dephosphorylation via Rap	100 h^{-1}
k_{10}	Spo0A$_{\mathrm{P}}$:Spo0E complex dissociation	100 h^{-1}
k_{11}	Spo0A$_{\mathrm{P}}$ dephosphorylation via Spo0E	100 h^{-1}
k_{pa}	Auto-phosphorylation of Spo0A	2 h^{-1}
k_{dpa}	Dephosphorylation of Spo0A$_{\mathrm{p}}$ and Spo0F$_{\mathrm{p}}$	0.05 h^{-1}
v_{kinA}^{0}	The basal transcription rate of *kinA*	0.9 μM/h
$v_{\mathrm{kinA}}^{\mathrm{max}}$	The maximal transcription rate of *kinA*	1.5 μM/h
K_{kinA}	Half-maximal binding constant for KinA expression	0.025μM
n_{kinA}	Hill-exponent for KinA expression	1
v_{0F}^{0}	The basal transcription rate of *spo0F*	0.15 μM/h
v_{0F}^{max}	The maximal transcription rate of *spo0F*	3 μM/h
K_{0F}	Half-maximal binding constant for Spo0F expression	0.15 μM
n_{0F}	Hill-exponent for Spo0F expression	2
v_{0B}	The transcription rate of *spo0B*	0.3 μM/h

v_{0A}^{0}	The basal transcription rate of *spo0A*	1.5 μM/h
v_{0A}^{max}	The maximal transcription rate of *spo0A*	6 μM/h
K_{0A}	Half-maximal binding constant for Spo0A expression	0.35 μM
n_{0A}	Hill-exponent for Spo0A expression	2
v_{Rap}	The transcription rate of *Rap*	0.075 μM/h
v_{0E}	The transcription rate of *spo0E*	0.03 μM/h
k_{d}	Rate constant of protein degradation	0.3 h^{-1}
k_{s}	Factor for delay effects	0.3 h^{-1}

The parameter values were adopted from Ref. (2).

References

1 Narula J, Devi SN, Fujita M, Igoshin OA. Ultrasensitivity of the *Bacillus subtilis* sporulation decision. *Proc. Natl. Acad. Sci. USA* 2012;109: E3513-3522.

2 Narula J, Kuchina A, Zhang F, Fujita M, Suel GM, Igoshin OA. Slowdown of growth controls cellular differentiation. *Mol. Syst. Biol.* 2016;12: 871.

3 Chai Y, Norman T, Kolter R, Losick R. Evidence that metabolism and chromosome copy number control mutually exclusive cell fates in *Bacillus subtilis*. *EMBO J.* 2011;30: 1402-1413.

4 Norman TM, Lord ND, Paulsson J, Losick R. Memory and modularity in cell-fate decision making. *Nature* 2013;503: 481-486.

5 Lord ND, Norman TM, Yuan R, Bakshi S, Losick R, Paulsson J. Stochastic antagonism between two proteins governs a bacterial cell fate switch. *Science* 2019;366: 116-120.

6 Chai Y, Kolter R, Losick R. Paralogous antirepressors acting on the master regulator for biofilm formation in *Bacillus subtilis*. *Mol. Microbiol*. 2009;74: 876-887.

7 Lehnik-Habrink M, Schaffer M, Mader U, Diethmaier C, Herzberg C, Stulke J. RNA processing in *Bacillus subtilis*: identification of targets of the essential RNase Y. *Mol. Microbiol*. 2011;81: 1459-1473.

8 Scott DJ, Leejeerajumnean S, Brannigan JA, Lewis RJ, Wilkinson AJ, Hoggett JG. Quaternary re-arrangement analysed by spectral enhancement: the interaction of a sporulation repressor with its antagonist. *J. Mol. Biol.* 1999;293: 997-1004.

9 Newman JA, Rodrigues C, Lewis RJ. Molecular basis of the activity of SinR protein, the master regulator of biofilm formation in *Bacillus subtilis*. *J. Biol. Chem*. 2013;288: 10766-10778.
